# Quantification of Fundus Autofluorescence Features in a Molecularly Characterized Cohort of >3500 Patients with Inherited Retinal Disease from the United Kingdom

**DOI:** 10.1016/j.xops.2024.100652

**Published:** 2024-11-12

**Authors:** William A. Woof, Thales A.C. de Guimarães, Saoud Al-Khuzaei, Malena Daich Varela, Sagnik Sen, Pallavi Bagga, Bernardo Mendes, Mital Shah, Paula Burke, David Parry, Siying Lin, Gunjan Naik, Biraja Ghoshal, Bart J. Liefers, Dun Jack Fu, Michalis Georgiou, Quang Nguyen, Alan Sousa da Silva, Yichen Liu, Yu Fujinami-Yokokawa, Dayyanah Sumodhee, Praveen Patel, Jennifer Furman, Ismail Moghul, Mariya Moosajee, Juliana Sallum, Samantha R. De Silva, Birgit Lorenz, Frank G. Holz, Kaoru Fujinami, Andrew R. Webster, Omar A. Mahroo, Susan M. Downes, Savita Madhusudhan, Konstantinos Balaskas, Michel Michaelides, Nikolas Pontikos

**Affiliations:** 1 University College London Institute of Ophthalmology, London, United Kingdom; 2 Moorfields Eye Hospital NHS Foundation Trust, London, United Kingdom; 3 Nuffield Laboratory of Ophthalmology, Nuffield Department of Clinical Neuroscience, University of Oxford, Oxford, United Kingdom; 4 Oxford Eye Hospital, John Radcliffe Hospital, Oxford, United Kingdom; 5 St Paul’s Eye Unit, Liverpool University Hospitals NHS Foundation Trust, Liverpool, United Kingdom; 6 Department of Ophthalmology and Epidemiology, Erasmus MC, Rotterdam, The Netherlands; 7 Laboratory of Visual Physiology, Division of Vision Research, National Institute of Sensory Organs, National Hospital Organization Tokyo Medical Center, Tokyo, Japan; 8 Department of Ophthalmology and Visual Sciences, Escola Paulista de Medicina, Federal University of Sao Paulo, Brazil; 9 Transmit Centre of Translational Ophthalmology, Justus-Liebig-University Giessen, Germany; 10 Department of Ophthalmology, University Hospital Bonn, Bonn, Germany

**Keywords:** Artificial intelligence, Fundus autofluorescence, Hyper-autofluorescence (Hyper-AF), Hypo-autofluorescence (Hypo-AF), Inherited retinal disease

## Abstract

**Purpose:**

To quantify relevant fundus autofluorescence (FAF) features cross-sectionally and longitudinally in a large cohort of patients with inherited retinal diseases (IRDs).

**Design:**

Retrospective study of imaging data.

**Participants:**

Patients with a clinical and molecularly confirmed diagnosis of IRD who have undergone 55° FAF imaging at Moorfields Eye Hospital (MEH) and the Royal Liverpool Hospital between 2004 and 2019.

**Methods:**

Five FAF features of interest were defined: vessels, optic disc, perimacular ring of increased signal (ring), relative hypo-autofluorescence (hypo-AF), and hyper-autofluorescence (hyper-AF). Features were manually annotated by 6 graders in a subset of patients based on a defined grading protocol to produce segmentation masks to train an artificial intelligence model, AIRDetect, which was then applied to the entire imaging data set.

**Main Outcome Measures:**

Quantitative FAF features, including area and vessel metrics, were analyzed cross-sectionally by gene and age, and longitudinally. AIRDetect feature segmentation and detection were validated with Dice score and precision/recall, respectively.

**Results:**

A total of 45 749 FAF images from 3606 patients with IRD from MEH covering 170 genes were automatically segmented using AIRDetect. Model-grader Dice scores for the disc, hypo-AF, hyper-AF, ring, and vessels were, respectively, 0.86, 0.72, 0.69, 0.68, and 0.65. Across patients at presentation, the 5 genes with the largest hypo-AF areas were *CHM*, *ABCC6*, *RDH12*, *ABCA4*, and *RPE65*, with mean per-patient areas of 43.72, 29.57, 20.07, 19.65, and 16.92 mm^2^, respectively. The 5 genes with the largest hyper-AF areas were *BEST1*, *CDH23*, *NR2E3*, *MYO7A*, and *RDH12*, with mean areas of 0.50, 047, 0.44, 0.38, and 0.33 mm^2^, respectively. The 5 genes with the largest ring areas were *NR2E3, CDH23*, *CRX*, *EYS*, and *PDE6B*, with mean areas of 3.60, 2.90, 2.89, 2.56, and 2.20 mm^2^, respectively. Vessel density was found to be highest in *EFEMP1*, *BEST1*, *TIMP3*, *RS1*, and *PRPH2* (11.0%, 10.4%, 10.1%, 10.1%, 9.2%) and was lower in retinitis pigmentosa (RP) and Leber congenital amaurosis genes. Longitudinal analysis of decreasing ring area in 4 RP genes (*RPGR*, *USH2A*, *RHO*, and *EYS*) found *EYS* to be the fastest progressor at −0.178 mm^2^/year.

**Conclusions:**

We have conducted the first large-scale cross-sectional and longitudinal quantitative analysis of FAF features across a diverse range of IRDs using a novel AI approach.

**Financial Disclosure(s):**

Proprietary or commercial disclosure may be found in the Footnotes and Disclosures at the end of this article.

Inherited retinal diseases (IRDs) are clinically and genetically heterogeneous disorders that affect the retina and represent the leading cause of legal blindness among working-age adults in England and Wales, and the second most common cause in childhood.^1^ This group of disorders can be caused by genetic variants in any 1 of more than 300 genes.^2^, ^3^, ^4^

Many IRDs are associated with structural changes within the retina, which can be detected with retinal imaging using different imaging modalities such as color fundus, infrared-reflectance, spectral-domain OCT, or fundus autofluorescence (FAF). Fundus autofluorescence is of particular importance in the context of IRDs because it allows the detection of patterns of fluorophores, often at the level of the photoreceptors and retinal pigment epithelium (RPE), which can be indicative of pathological changes such as loss of overlying photoreceptors.^5^^,^^6^ Some of these FAF signal changes are highly characteristic of specific IRDs and can indicate features such as areas of RPE atrophy or lipofuscin deposits. Fundus autofluorescence is listed as a primary or secondary outcome in multiple clinical trials, and it has become a useful retinal biomarker for diagnostic and prognostication purposes in a wide variety of IRDs.^3^^,^^5^^,^^7^^,^^8^

The identification and quantification of disease-associated features within retinal imaging are critical for diagnosis, monitoring disease progression, providing prognostic information, and assessing treatments in IRDs. The first step in quantifying retinal imaging-based biomarkers of disease involves the identification and segmentation of these features. Manual segmentation performed by human annotators is time-consuming and requires expert annotators, which makes this process subjective and not feasible on a large scale. Automated identification and segmentation of IRD features in a reliable way is important for enabling the routine use of these data quantitatively in clinical practice and helping further our understanding of these diseases.

Existing studies that have used deep learning to segment IRD features from retinal images have so far focused on specific IRD phenotypes such as retinitis pigmentosa (RP), Stargardt (STGD1), and choroideremia (CHM).^9^^,^^10^

To support our analysis of a broad range of different IRD phenotypes, we developed AIRDetect, a deep-learning model that can automatically identify and segment relevant features from FAF images. We apply AIRDetect to a large cohort of patients with IRD with molecularly confirmed diagnoses at Moorfields Eye Hospital (MEH), to identify genotype–phenotype associations, as well as quantify disease progression.

## Methods

### Data Set Curation

Patients’ genotypes were extracted from the Genetics database of MEH (London, United Kingdom).^2^^,^^11^ Patients’ images were exported from the Heidelberg Imaging (Heyex) database (Heidelberg Engineering) based on their hospital number, for records between June 17, 2004, and October 22, 2019. All 55-degree FAF images were 488 nm blue-FAF images captured by the Heidelberg Spectralis and the HRA2 imaging platforms.

A data set of 736 blue-FAF images (55°) from 573 patients from MEH was annotated with 4 different image features, optic disc, regions of hyper-autofluorescence (hyper-AF) and hypo-autofluorescence (hypo-AF), and perimacular ring of increased signal, and a further set of 206 blue-FAF images (55°) from 127 patients from the Royal Liverpool Hospital (RLH) were annotated with the retina vessel tree. A grading protocol was defined for IRD retinal feature annotations (Table 1).^12^, ^13^, ^14^ The Dice similarity coefficient score was used to assess intergrader agreement.^15^ The Dice similarity coefficient is defined as twice the area of overlap between 2 annotations divided by the total area occupied by the 2 annotations. It ranges from 1 for perfect overlap between 2 annotations to 0 for no overlap between 2 annotations. The intergrader agreement was not found to be significantly different between the graders. Manual grading was completed over an 18-month period from June 2022 to December 2023 by 4 graders, with 2 additional graders carrying out the vessel segmentation at RLH. The 4 MEH graders were research fellows with >5 years of experience in medical retina, 3 of whom had 3 years of experience with FAF scans and IRDs. The 2 RLH graders were staff from the RLH Reading Centre with >5 years of experience in vessel annotation on FAF scans. Manual grading was performed using the Moorfields Grading Portal online platform (grading.readingcentre.org). A full breakdown of the manually annotated data set is given in Table S2 (available at www.ophthalmologyscience.org).

**Table 1:** Features and Definitions Used during the Annotation Process of 5 Features by the Graders

Name	Shorthand	Includes	Excludes
Optic disc	disc	The optic nerve head. Includes both the optic cup and rim.	Peripapillary atrophy not included in the annotation.
Hypo-autofluorescence	hypo-AF	Areas distinctly darker than physiological normal areas with 50% grader confidence. The level of hypo-AF should be at least 90%–100% as dark as the optic disc. This is defined as definitely decreased AF (DDAF) in the literature.^14^^,^^15^ Note this is relative AF rather than absolute AF.	Excludes peripapillary atrophy. Areas of ambiguous (not definitely decreased) regions in the periphery are not annotated as hypo-AF.
Hyper-autofluorescence	hyper-AF	Regions brighter than the physiological normal area with 50% grader confidence. Note this is relative AF rather than absolute AF.	Excludes macular ring. Excludes flecks.
Perimacular ring of increased signal	ring	Ring-shaped area of hyper-AF within the vascular arcades at the macula.	Must be >50% complete circle.
Veins and arteries	vessels	All visible retinal vessels stemming from the optic disc.	Only annotated over atrophy if the grader is >50% certain of the location of the vessel.

### Training and Test Data Sets

The annotated data set was compiled, and any images without confirmation for all features from ≥1 grader at the time of model development were discarded; to avoid bias, the annotation from a single grader was randomly selected where multiple grader annotations were available for a single image. After this process, there were 554 images from 464 patients from MEH. The MEH training set consisted of 506 images from 424 patients. The MEH hold-out test set consisted of 48 images from 40 patients. The RLH training set consisted of 72 images from 52 patients from RLH. The RLH hold-out test set consisted of 23 images from 22 patients. Training sets were split into 5 separate sets for use with fivefold cross-validation, ensuring a balanced representation of each class across folds. Assignment to the training and test sets was done at the patient-level to avoid any potential data leakage. The data flowchart is fully described in Figure S1 (available at www.ophthalmologyscience.org).

### Development of AIRDetect Segmentation Model

We developed AIRDetect, a deep-learning segmentation algorithm, to automatically identify and segment the chosen features from FAF images. For training the neural network we selected the no-new-UNet (nnU-Net) framework for its adaptability and performance in automatic medical image segmentation tasks.^16^ At its core, nnU-Net leverages a fully convolutional network design inspired by the U-Net architecture, renowned for its efficacy in medical imaging tasks.^17^, ^18^, ^19^ The overlying nnU-Net framework then automatically configures its network architecture, preprocessing, and training strategy based on the data set's characteristics, optimizing for performance, without requiring manual hyperparameter tuning or architecture modifications from the user.

For the 5 different image features, we trained 2 separate nnU-net models. A single multiclass model for disc, hyper-AF and hypo-AF, and ring, and a separate single-class model for vessels. As with common practice for nn-Unet, each model consisted of an ensemble of 5 U-nets with identical architectures, but different weights, trained independently and then ensembled at inference, taking the unweighted average of the probability scores across networks.

The model was trained using a sum of Dice and cross-entropy loss functions to optimize for multiclass segmentation accuracy. Hyperparameters, such as learning rate and batch size, were selected by the nnU-Net based on its analysis of the data set. Training was curtailed at 200 epochs as this was sufficient to achieve convergence in most cases.

### Validation of AIRDetect Segmentation Model

Model validation was assessed using the Dice coefficient between the model predictions and the corresponding grader annotation on the hold-out test set. Where images were double-graded, we took the mean of the model-grader Dice for each grading. We also analyzed the accuracy of the model-grader agreement for simple presence/absence detection where we counted cases as positive for which the model/annotator marked at least some part of the image for the given feature, and negative otherwise, from which we derived presence/absence detection accuracy, precision, and recall.

### Automatic Annotations on Data Set from IRD Clinics

The trained models were applied to automatically segment 45 749 FAF images (55°) from 3606 patients with IRD with a molecularly confirmed diagnosis from MEH covering 170 genes.^2^^,^^11^ This took on average 1 second per image parallelized over four 3090 Nvidia GPUs amounting to approximately 3 to 4 hours in total. In comparison, a human grader could take 5 to 30 minutes per scan amounting to 2 to 12 years full-time equivalent (assuming 8 hours a day, 5 days a week) in total. Images where the optic disc was not segmented by the model were removed, because these images were of poor quality or not centered on the macula (Fig S2, available at www.ophthalmologyscience.org). Results were analyzed from 33 042 FAF images from 3496 patients, after filtering.

For each of the generated masks, we extracted (1) if the feature was present or absent; (2) the area, the number of pixels in the segmented mask multiplied by the resolution; (3) the number of connected components, found using watershed clustering^20^; and (4) feature brightness, mean intensity of pixels from the region covered by the segmented mask. For vessels, we calculated a selection of metrics defined in Table S3 (available at www.ophthalmologyscience.org), using the provided code from the reti-py library as used in the AutoMorph repository.^21^ Features were also analyzed based on their distance from the fovea.

To calculate the rate of progression for a given feature, a linear regression was fit to each patient's eye, taking time since the first appointment (in years) as the independent variable, and taking the calculated areas of the segmented feature at each time-point as the measured variable. The slope of the regression was then averaged across eyes per patient to give a rate of progression. When multiple scans per eye were present for a given date, we took the most recent scan with the rationale that good-quality scans were less likely to lead to further imaging by the operator.

## Results

### AIRDetect Model Validation

**Figure 3:**
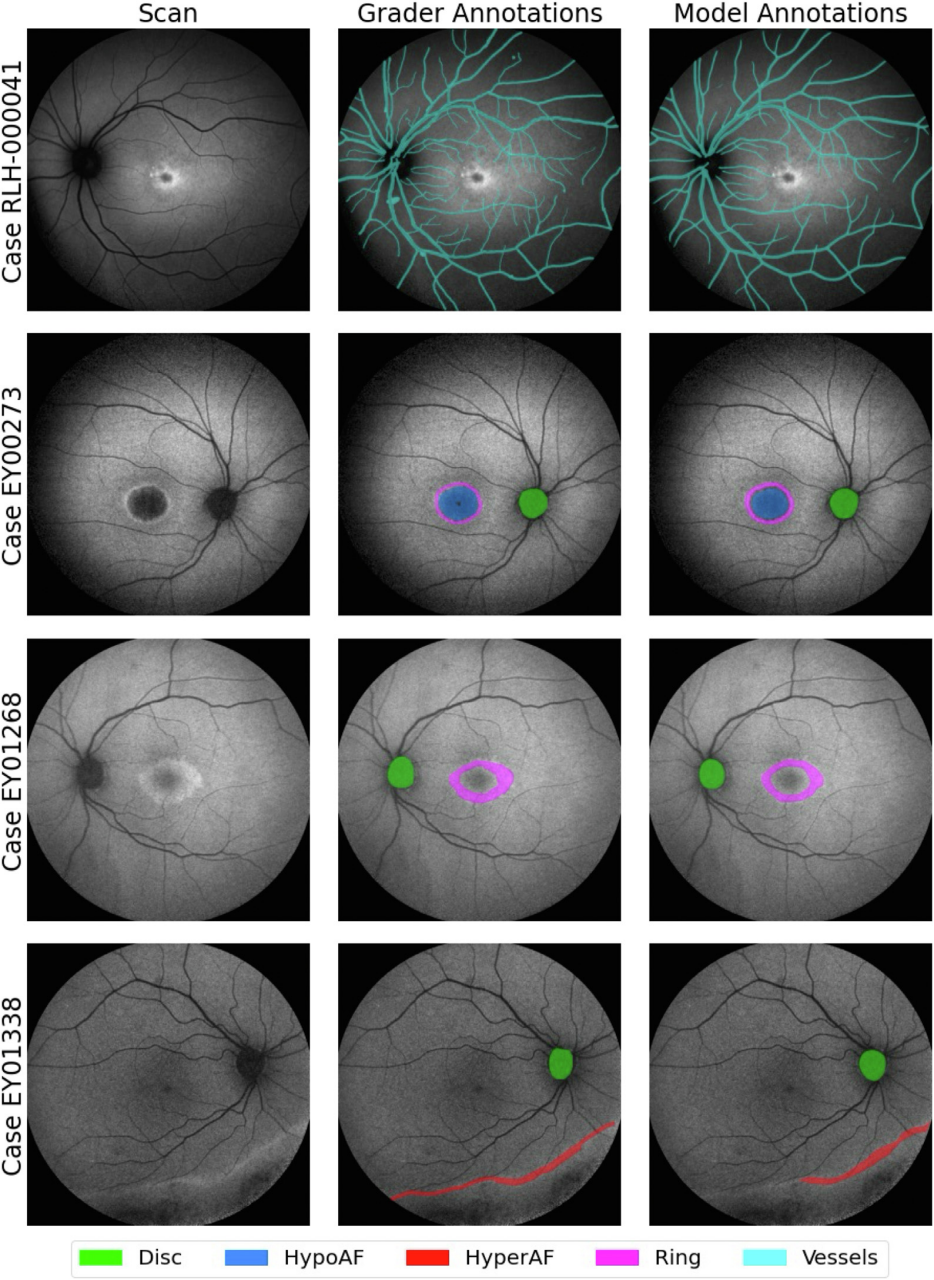
Examples of manually and automatically segmented masks for the 5 features: vessels, disc, ring, hyper-autofluorescence (hyper-AF), and hypo-autofluorescence (hypo-AF). The vessel data set was separate from the rest of the data; hence, vessel visualization is separate from other features.

Examples of AIRDetect segmentation output are presented in Figure 3. Model-grader Dice scores for disc, hypo-AF, hyper-AF, ring, and vessels were respectively 0.86, 0.72, 0.69, 0.68, and 0.65, with intergrader Dice scores of 0.82, 0.75, 0.72, 0.80, and 0.95, respectively. Model detection accuracy ranged from 77% to 83% (excluding anatomical features) (Table 4). Features which were the most challenging to detect were hyper-AF and ring as those had the lowest precision scores at 0.53 and 0.60, respectively.

**Table 4:** Segmentation Model Training Data and Results. Dice Score Quantifies the Model's Segmentation Performance and Presence/Absence Quantifies its Feature Detection Performance

Feature	Train Set		Test Set		Segmentation (Dice)		Detection (Presence/Absence)		
	Total	Incidence	Total	Incidence	Intergrader	Model-Grader	Accuracy	Precision	Recall
Disc	506	98%	48	98%	0.82	0.86	–	–	–
Hypo-AF	506	70%	48	44%	0.75	0.72	83.3%	0.81	0.81
Hyper-AF	506	18%	48	23%	0.72	0.69	79.2%	0.53	0.82
Ring	506	32%	48	31%	0.80	0.68	77.1%	0.60	0.80
Vessels	72	100%	23	100%	0.94	0.65	–	–	–

Dice intergrader = intergrader agreement of double-graded images; Dice model-grader = Dice score between model and graders, with mean scores, used when images were double-graded; Incidence = percent of images with gradable feature; Total = number of annotated images.

### Genotype-–Phenotype Associations

**Figure 4:**
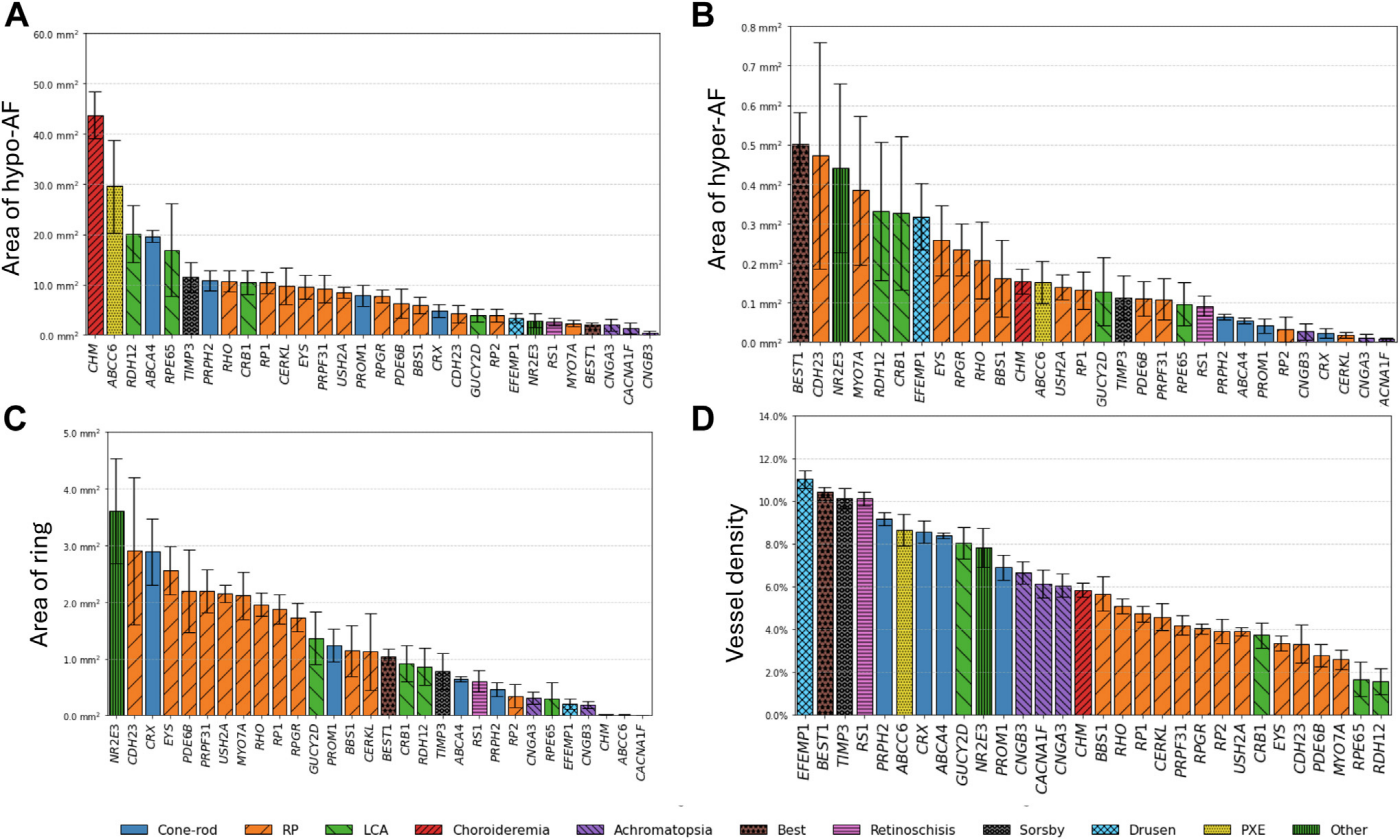
Bar plot representing **A,** area of hypo-autofluorescence (hypo-AF); **B,** area of hyper-autofluorescence (hyper-AF); **C,** area of ring; and, **D,** vessel density (ratio between area of vessels and total image area) across the 30 most common genes (*RPE65* included for reference). Error bars denote standard error. Values were measured from images at first presentation before averaging by gene. Genes are grouped into approximate phenotype groupings denoted by bar styling.

Analyzing associations between identified features and genes across most common genes (Table S5, available at www.ophthalmologyscience.org), the 5 genes with the largest hypo-AF areas at first presentation were *CHM*, *ABCC6*, *RDH12*, *ABCA4*, and *RPE65*, with mean per-patient areas of 43.72, 29.57, 20.07, 19.65, and 16.92 mm^2^, respectively (Fig 4A). *CHM* exhibited the largest hypo-AF areas across all age ranges, with the exception of the over 45-year-old group, in which *RPE65* was the highest (Fig S5, available at www.ophthalmologyscience.org). The 5 genes with the largest hyper-AF areas were *BEST1*, *CDH23*, *NR2E3*, *MYO7A*, and *RDH12*, with mean areas of 0.50, 0.47, 0.44, 0.38, and 0.33 mm^2^, respectively (Fig 4B). At presentation, *CDH23 and BEST1* exhibited the largest hyper-AF area in the under 18-year-olds, *MY07A* and *BEST1* in the 18- to 30-year-olds, *CRB1*, *RDH12*, and *BEST1* in the 30- to 45-year-olds, and *NR2E3* and *CDH23* in the over 45-year-olds (Fig S6, available at www.ophthalmologyscience.org). The 5 genes with the largest ring areas at first presentation were *NR2E3*, *CDH23*, *CRX*, *EYS*, and *PDE6B*, with mean areas of 3.60, 2.90, 2.89, 2.56, and 2.20 mm^2^, respectively (Fig 4C). *RPGR* and *MYO7A* exhibited the largest ring areas in those aged <18 years, *PRPF31*, *USH2A*, and *RHO* in those aged 18 to 30 years, and *CDH23* and *NR2E3* in those aged >30 years (Fig S7, available at www.ophthalmologyscience.org).

At the gene variant level, *ABCA4* p.(Gly1961Glu) showed a higher ring area than other common *ABCA4* variants (Fig S8, available at www.ophthalmologyscience.org). Vessel density was found to be highest in *EFEMP1*, *BEST1*, *TIMP3*, *RS1*, and *PRPH2* (11.0%, 10.4%, 10.1%, and 9.2%) and was lower in RP and Leber congenital amaurosis associated genes (Fig 4D). A full breakdown of features across the 30 most common genes is given in Table S5, for all genes in Table S6 (available at www.ophthalmologyscience.org), and for vessels in Table S7 (available at www.ophthalmologyscience.org). To account for the potential differences in age of onset and disease stage between genes, we have produced a full breakdown per age group in Figures S5, S6, and S7 (available at www.ophthalmologyscience.org).

**Figure 9:**
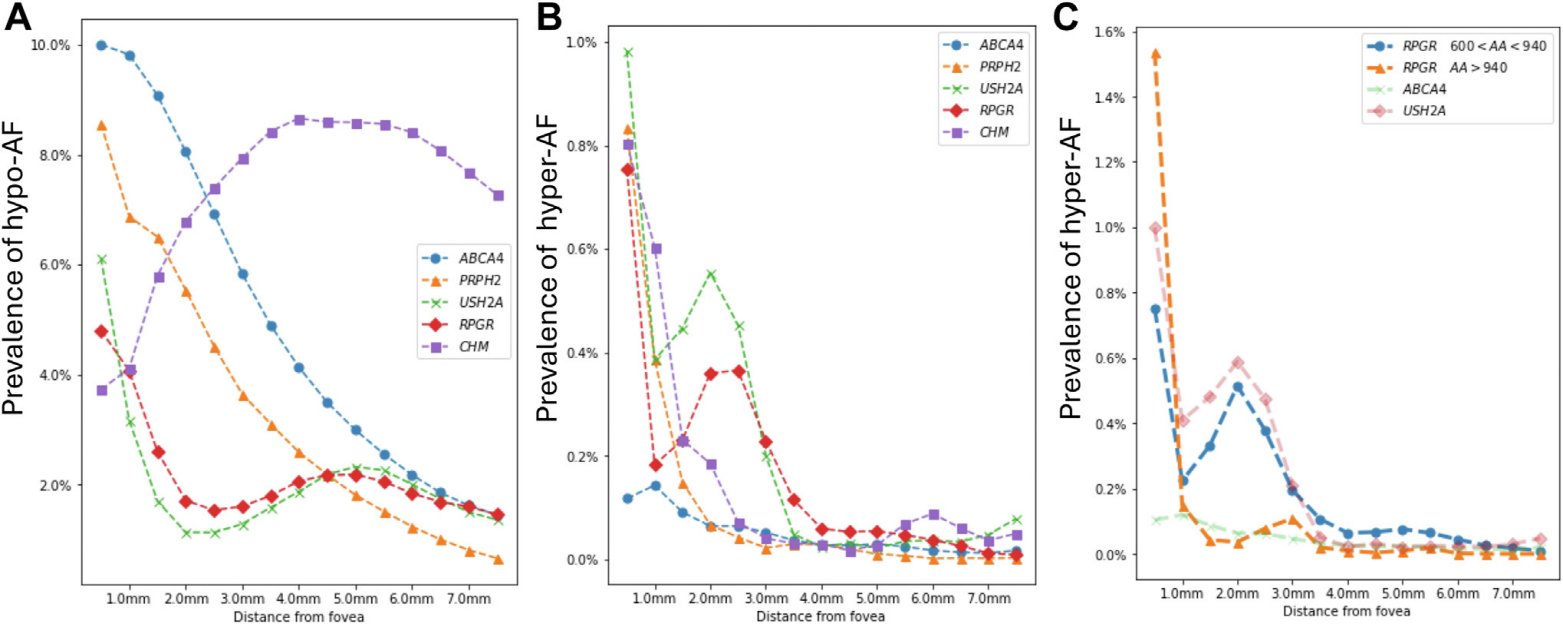
Autofluorescence (AF) as a proportion of total altered AF area in the image compared with distance from fovea for patients with variants in *ABCA4*, *RPGR*, *USH2A*, *RPGR*, and *CHM* for **A,** hypo-AF and **B,** hyper-AF. **C,** Difference in phenotype according to RPGR region illustrated by the prevalence of hyper-AF from the fovea. Between peptide positions 600 and 940, the phenotype is more similar to rod–cone phenotype (as *USH2A*), whereas after position 940 it tends to be more similar to a cone–rod phenotype (as *ABCA4*).

We analyzed how features vary with distance from the fovea by looking at the prevalence of each feature in each 0.5 mm annulus moving away from the fovea. Figure 9 compares the prevalence of hyper- and hypo-AF at different distances from the fovea in 5 different genes (see Fig S10, available at www.ophthalmologyscience.org for scale), as well as between regions within 1 gene (Fig 9C). The 2 genes associated largely with maculopathy or cone–rod dystrophy (*ABCA4* and *PRPH2*) show increased area and prevalence of hypo-AF at the fovea (Fig 9A) but reducing proportions of the retina displaying hypo-AF moving away from the fovea. The 2 RP-associated genes (*USH2A* and *RPGR*) show less hypo-AF across the central 55° of the retina compared with the cone–rod genes, but with a bimodal profile, with the greatest relative proportion of hypo-AF at the fovea followed by 4 to 6 mm from the fovea, just within the vascular arcade. For *CHM*, unlike the other genes, there was the least hypo-AF at the fovea, but substantially increased hypo-AF away from the fovea. For hyper-AF, there is an increased proportion of hyper-AF at the fovea in all genes except *ABCA4* which reduces further from the fovea (Fig 9B). In the 2 RP-associated genes (*USH2A* and *RPGR*) there is an increase in hyper-AF at 1 to 3 mm from the fovea. In Figure 9C, we apply a similar visualization but only consider 2 regions in RPGR.^22^ The first region includes variants that affect the peptide sequence from amino acid 600 to 940, and the second region includes variants that affect the peptide sequence downstream of amino acid 940. In the first region, the *RPGR* phenotype tends to be more similar to rod–cone dystrophies such as *USH2A*, whereas, if the second region is affected, the phenotype tends to be more similar to cone–rod dystrophies such as *ABCA4*.

**Figure 11:**
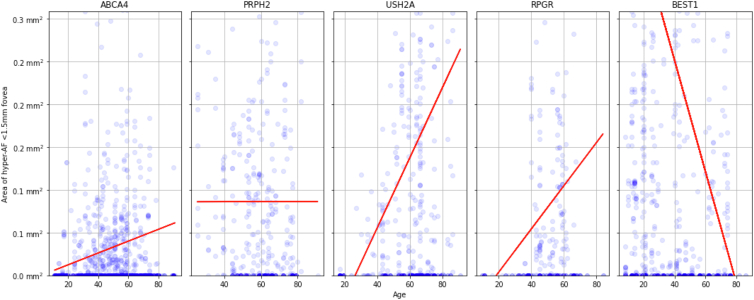
Hyper-autofluorescence (hyper-AF) area within 1.5 mm of the fovea (corresponding to inner 3 mm ETDRs ring) compared with patient age. The least-squares regression line indicated in red. Significant increase in hyper-AF with age for *ABCA4* (β = 691 μm^2^/year; *P* < 0.001), *USH2A* (β = 4090 μm^2^/year; *P* < 0.001), and *RPGR* (β = 2520 μm^2^/year; *P* < 0.029). Significant decrease for *BEST1* (β = –6500 μm^2^/year; *P* < 0.001). No significant changes of hyper-AF with age were found for *PRPH2*.

In Figure 11, the area of hyper-AF within 1.5 mm of the fovea is compared against patient age for 5 different IRD genes. Most genes showed an increase with age, with the exception of *PRPH2*, which remained fairly stationary, and *BEST1*, which demonstrated a sharp decrease with patient age, although there was considerable variability across ages within all genes.

### Disease Progression

**Figure 12:**
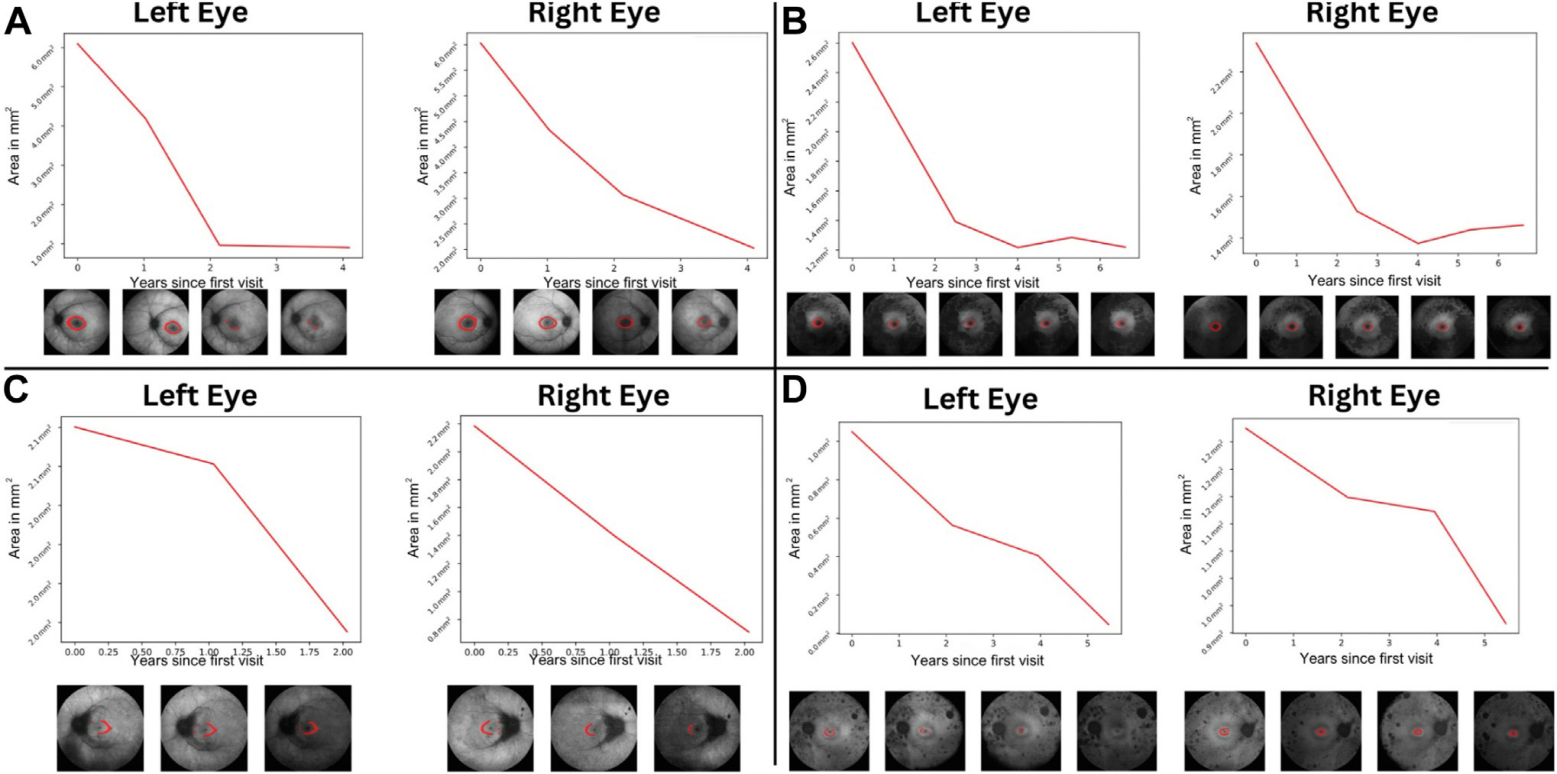
Automatic monitoring of lesion size for disease progression. Decreasing area of the ring for 4 patients with disease-causing variants in: **A,***RPGR*; **B,***USH2A*; **C,***RHO*; and **D,***EYS*. In these genes, the macular ring is expected to shrink in diameter over time as the disease progresses.

We applied AIRDetect longitudinally to monitor progression within individual patients across multiple visits. Figure 12 shows an example using AIRDetect to visualize the decrease in ring area in individual patients with RP associated with variants in 4 different genes, namely *USH2A*, *PRPH2*, *RHO*, and *EYS*. This type of analysis was previously undertaken in other IRD cohorts.^23^, ^24^, ^25^, ^26^ Comparing these 4 RP genes in the entire MEH IRD cohort, the average rate of decrease in total ring area was greater in patients with RP associated with *EYS* (−0.178 mm^2^/year; *n* = 40; standard deviation [SD] = 0.857), *USH2A* (−0.066 mm^2^/year; *n* = 245; SD = 1.040), and *RPGR* (−0.046 mm^2^/year; *n* = 115; SD = 0.554), when compared with *RHO* (−0.040 mm^2^/year; *n* = 73; SD = 0.458).

**Figure 13:**
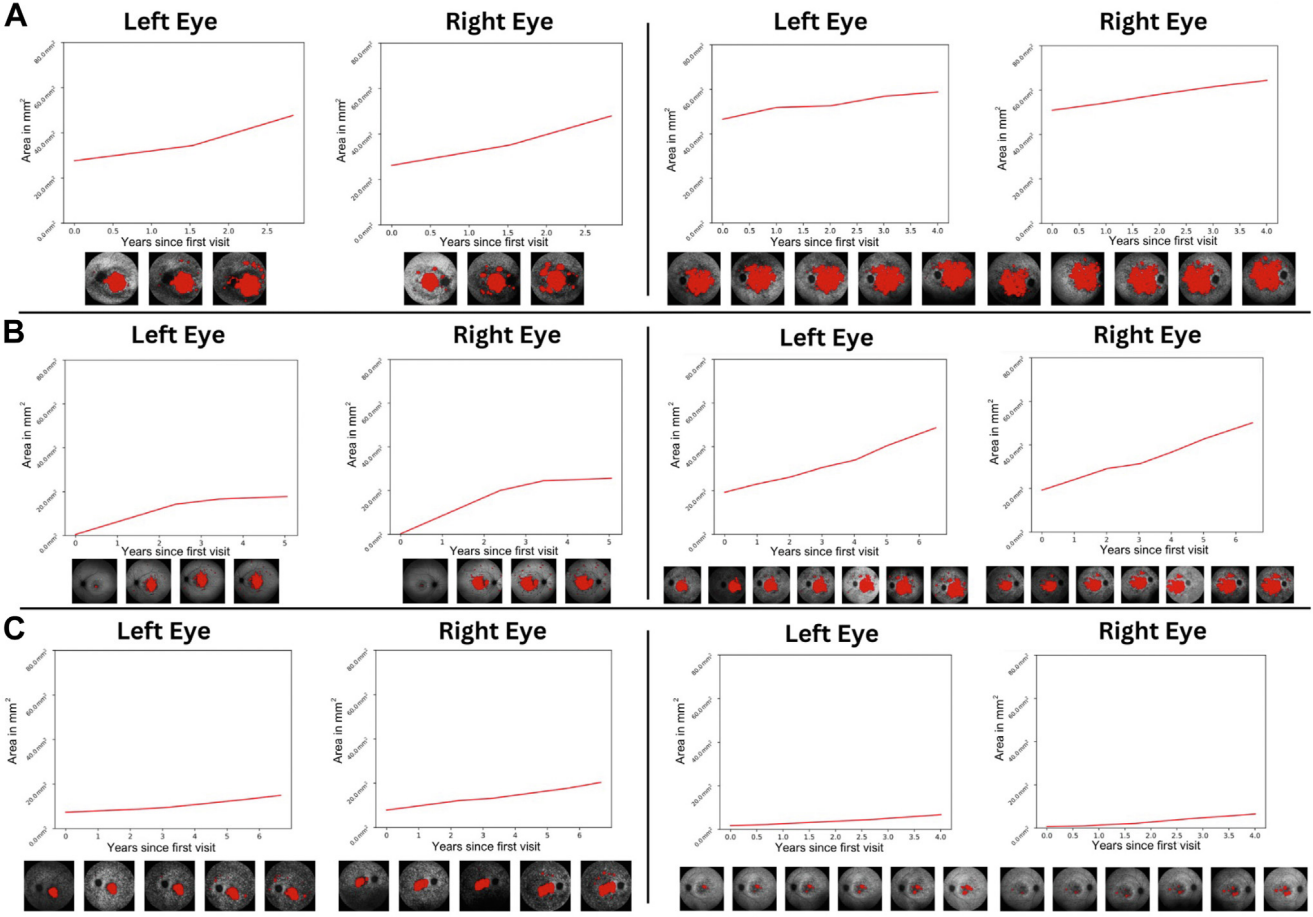
Increasing area of hypo-autofluorescence for 2 patients of each of the 3 *ABCA4* severity groups: **A,** group A; **B,** group B; and **C,** group C. Here we see the expected patterns of progression reported in Table S8 with A being the fast progressors, followed by B and C.

We also applied AIRDetect to monitor progression in patients belonging to 3 subgroups of *ABCA4* (Fig 13). Patients were classified into 3 groups (A, B, and C) based on the increasing severity of genetic variants as defined by Cornelis et al.^27^^,^^28^ Patients in group A had 2 severe variants, whereas group C had a mild variant in trans with any other variant. Patients with variants of known severity whose combination does not fit the other 2 groups were placed into group B. The average increase in hypo-AF area per year was compared between groups (Fig S14, available at www.ophthalmologyscience.org). In keeping with previous studies,^25^^,^^29^, ^30^, ^31^, ^32^ the mean per-patient rate of increase in hypo-AF area was highest in the highest severity classification (group A), at 3.11 mm^2^/year (*n* = 69; SD = 5.80), followed by 1.59 mm^2^/year (*n* = 75; SD = 6.74) for the intermediate severity group (B), and finally 0.87 mm^2^/year (*n* = 184; SD = 3.13) in the lowest severity group (C) (Table S8, available at www.ophthalmologyscience.org).

## Discussion

The results of our cross-sectional analysis match known genotype–phenotype associations demonstrating the validity of our approach, as well as yielding novel insights. For example in Figure 4A, *CHM* and *ABCA4* both exhibited high levels of hypo-AF, consistent with the large areas of atrophy that spare the fovea in CHM, as well as the macular atrophy typically seen in STGD1 disease (*ABCA4*).^7^^,^^33^, ^34^, ^35^ Of interest, however, *ABCC6*, which is associated with pseudoxanthoma elasticum was identified to have the second largest areas of hypo-AF. On further inspection, these could be explained by the large angioid streaks characteristic of this condition, which can appear as hypo-AF on FAF.^36^ For hyper-AF, *BEST1* exhibited the largest areas of hyper-AF, which can be attributed to the vitelliform lesion(s) that are characteristically observed in autosomal dominant and recessive forms of the disease (Fig 4B).^37^, ^38^, ^39^ For ring, the presence of a macular ring typically corresponds to a demarcation between the diseased and nondiseased retina and is usually seen in RP and cone–rod dystrophies, in keeping with our findings here (Fig 4C).^3^ The lower vessel density observed in RP and Leber congenital amaurosis genes was also in keeping with the vessel attenuation commonly associated with these genes^40^, ^41^, ^42^, ^43^(Fig 4D). As well as genotype–phenotype associations, we also found associations at the individual variant level confirming the known association between the p.(Gly1961Glu) variant in *ABCA4* and the presence of a macular ring (Fig S7).^44^, ^45^, ^46^, ^47^ When considering feature prevalence from the fovea, we found, as expected, that genes usually associated with cone–rod degeneration showed a decrease in hypo-AF extent moving away from the fovea but with an opposite trend for the RP genes and *CHM* (Fig 9A). Hyper-AF was mainly concentrated at the fovea, but with a distinctive peak at 2 to 3 mm from the fovea which may be attributed to partial macular rings classified as hyper-AF by our model (Fig 9B). *PRPH2* also had a higher coverage of hyper-AF in the fovea when compared with *ABCA4*, which is consistent with the pattern/macular dystrophy and adult vitelliform phenotypes associated with *PRPH2* (Fig 9B).^48^

In our longitudinal analysis, we were able to replicate the findings of Fakin et al^25^ 2016 in Figure 13 and Table S8, in which we found that the growth of areas of hypo-AF was much more rapid in the group associated with more severe *ABCA4* genetic variants.^25^ Our estimates for the rate of progression were higher than that previously reported, which may be due to the use of 55° as opposed to 30° imaging in our data set, hence a larger area of hypo-AF.^31^^,^^49^ Comparing hyper-AF across patient age in Figure 11, the hyper-AF within 1.5 mm of the fovea increased for *ABCA4*, *USH2A*, and *RPGR*, consistent with lesions developing with disease progression over time. However, there were some noteworthy exceptions for individual genes. In particular, *BEST1* is associated with “yolk-like” regions of hyper-AF, typically within 2 to 3 mm of the perifovea, which change over time through previtelliform, vitelliform, pseudohypopyon, and vitelliruptive stages and finally to the atrophic stage.^3^^,^^39^ The highest hyper-AF signal would be associated with the vitelliform stage, progressively reducing in intensity to become a region of hypo-AF by the atrophic stage, which matches what we see as a decrease in foveal hyper-AF with age. No significant progression of hyper-AF with age was detected for *PRPH2,* which is likely due to the later onset of the condition in most patients (typically, after 45 years of age) and hence a more limited age range, as well as a milder pattern of dystrophy.^50^

We also identified an increased rate of decrease in the area of the macular ring in *EYS*, *USH2A*, and *RPGR* compared with *RHO* (Fig 12). Monitoring the rate at which the macular ring narrows down is common practice in generalized retinal dystrophies such as RP.^5^ A more rapid encroachment of the macular ring in autosomal recessive (*USH2A* and *EYS*) and X-linked (*RPGR*) genes compared with the autosomal dominant *RHO* is consistent with the latter having a slower disease progression compared with the others.^51^

To date, deep-learning AI models to analyze FAF images from IRD patients have been limited. There have been studies developing classification models of FAF images based on IRD phenotypes.^52^, ^53^, ^54^, ^55^ However, in regards to segmentation approaches, areas of hypo-AF have been measured either manually or semiautomatically using RegionFinder on HEYEX2 software to study the progression rate of the area of atrophy in STGD1 disease.^56^, ^57^, ^58^, ^59^ These approaches compared with deep-learning approaches would be challenging to scale accurately to our real-world data set as they require considerable parameter tuning compared with deep-learning–based approaches such as AIRDetect. Previous deep-learning–based segmentation approaches have mostly focused on STGD1 to segment for hypo-AF^60^ or flecks.^10^ Hence, our AIRDetect approach represents the first to be developed and applied to a wide range of IRDs covering 170 genes.

One limitation of our approach is that the gene associations described in our study are limited by the variation in phenotypes, which can occur with the stage of disease for progressive conditions, different variants in the same gene, or different modes of inheritance. In terms of examples of phenotype variability per gene, *CRX* can be associated with a mild CORD but also quite severe Leber congenital amaurosis.^61^, ^62^, ^63^ *RPGR* can be associated with RP, Leber congenital amaurosis, macular dystrophy, and CORD.^23^^,^^64^ We conducted a subanalysis in *ABCA4* (Fig S7, available at www.ophthalmologyscience.org) but have not yet conducted this analysis across all gene variants and modes of inheritance.

Other limitations are the limited sample size for some of the genes and the large variance in imaging quality in our real-world data set in part due to the discomfort of the patient to potential blue-light toxicity,^65^ which affects the reliability of some of the features in lower quality images. Although automatic image quality assessment tools exist for color fundus retinal imaging,^66^ none have been developed for FAF imaging. Assessing image quality can also be particularly challenging for IRDs because they are associated with a wide range of pathologies, many of which can affect perceived image quality, as well as make it more challenging for the operator to acquire good-quality images. An IRD FAF image quality assessment model is under development, which should help to improve the consistency of our segmented masks and reduce noise in our analysis.^67^

We are also limited in our analysis to what is visible within the imaging, meaning we are only able to identify changes within the central 55° of the retina but not able to identify changes occurring in more peripheral retinal locations, which can be visible on ultra-widefield imaging.^68^ Differences in axial length can affect absolute measurements from retinal images. However, the refractive error is corrected during image acquisition because axial length information was unavailable in this study. It is possible that this can impact the accuracy of the measurements from the 55° FAF images, although the effect is expected to be limited in our analysis.

We anticipate that AIRDetect can be used to validate further clinically relevant findings, as well as identify new potential associations between different feature patterns and certain genes or variants. Our approach could also be applied to identifying structure–function association (Fig S15, available at www.ophthalmologyscience.org) as well as cross-modality image registration tasks by using vessel-based segmentation to align images (Fig S16, available at www.ophthalmologyscience.org). Besides IRDs, the diverse nature of IRD-associated pathologies might make AIRDetect useful to improve robustness for segmentation of FAF imaging for other non-IRD conditions or provide a good starting point for developing models for specific conditions, in which data are scarcer or to other imaging modalities such as ultra-widefield imaging, via transfer learning.

In conclusion, we have conducted, to our knowledge, the largest quantitative cross-sectional and longitudinal analysis of FAF features across a diverse range of IRDs in a real-world data set, enabled by our novel automatic segmentation AI model, AIRDetect.
