# Supplementary material for: Quantification of Fundus Autofluorescence Features in a Molecularly Characterized Cohort of >3500 Patients with Inherited Retinal Disease from the United Kingdom
Source: Ophthalmol Sci. 2024 Nov 12;5(2):100652. doi: 10.1016/j.xops.2024.100652 (PMC11782848; doi:10.1016/j.xops.2024.100652)

Figure S1: Data flowchart with number of images, patients, eyes, and genes at each stage of AIRDetect model development.

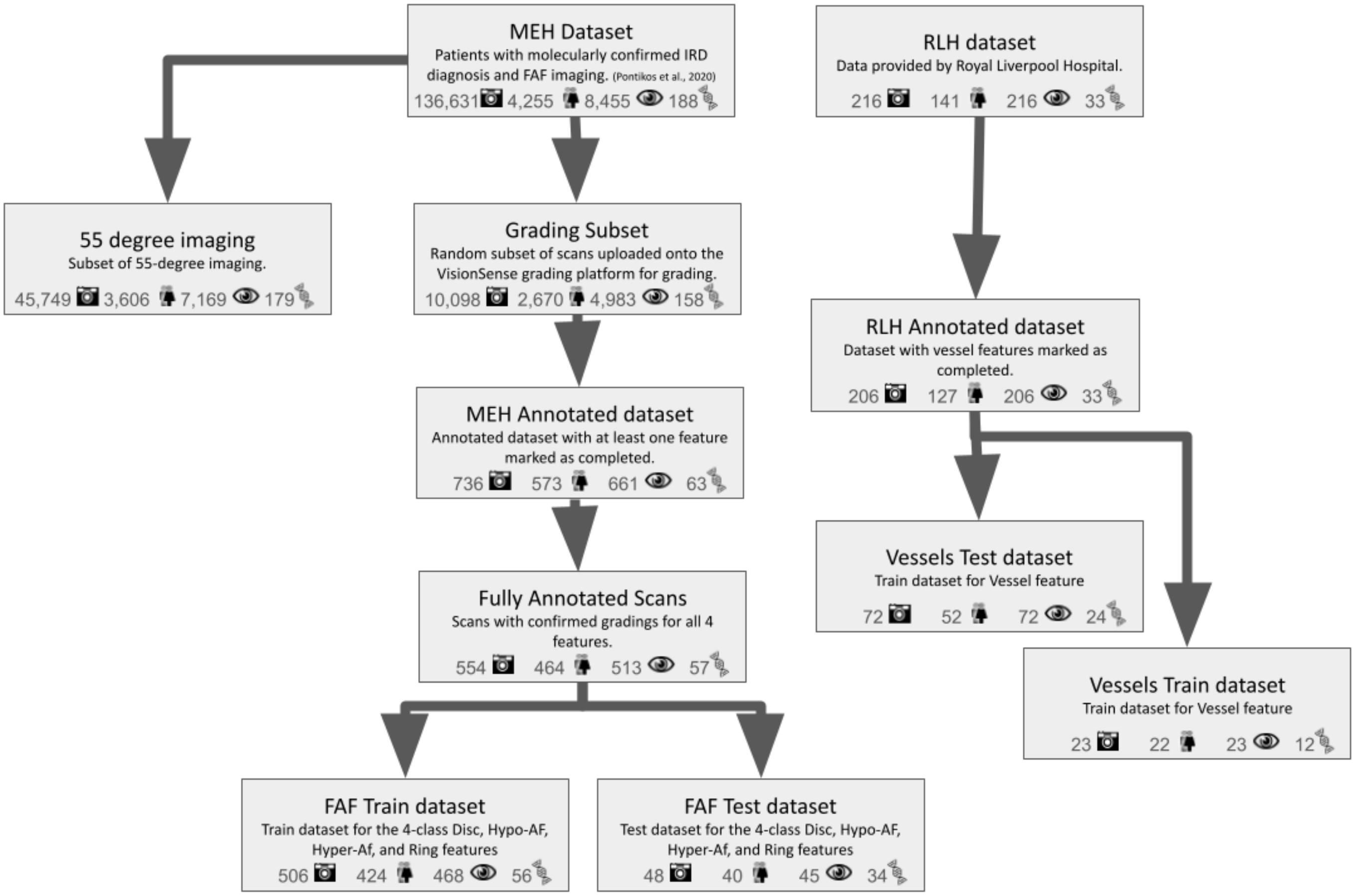

Supplement: Figure S1 [file mmc1.pdf]
