# Supplementary material for: Quantification of Fundus Autofluorescence Features in a Molecularly Characterized Cohort of >3500 Patients with Inherited Retinal Disease from the United Kingdom
Source: Ophthalmol Sci. 2024 Nov 12;5(2):100652. doi: 10.1016/j.xops.2024.100652 (PMC11782848; doi:10.1016/j.xops.2024.100652)

Figure S2: Examples of images with no Disc segmentation from the model. These are either poor quality, have significant atrophy, or are improperly centred.

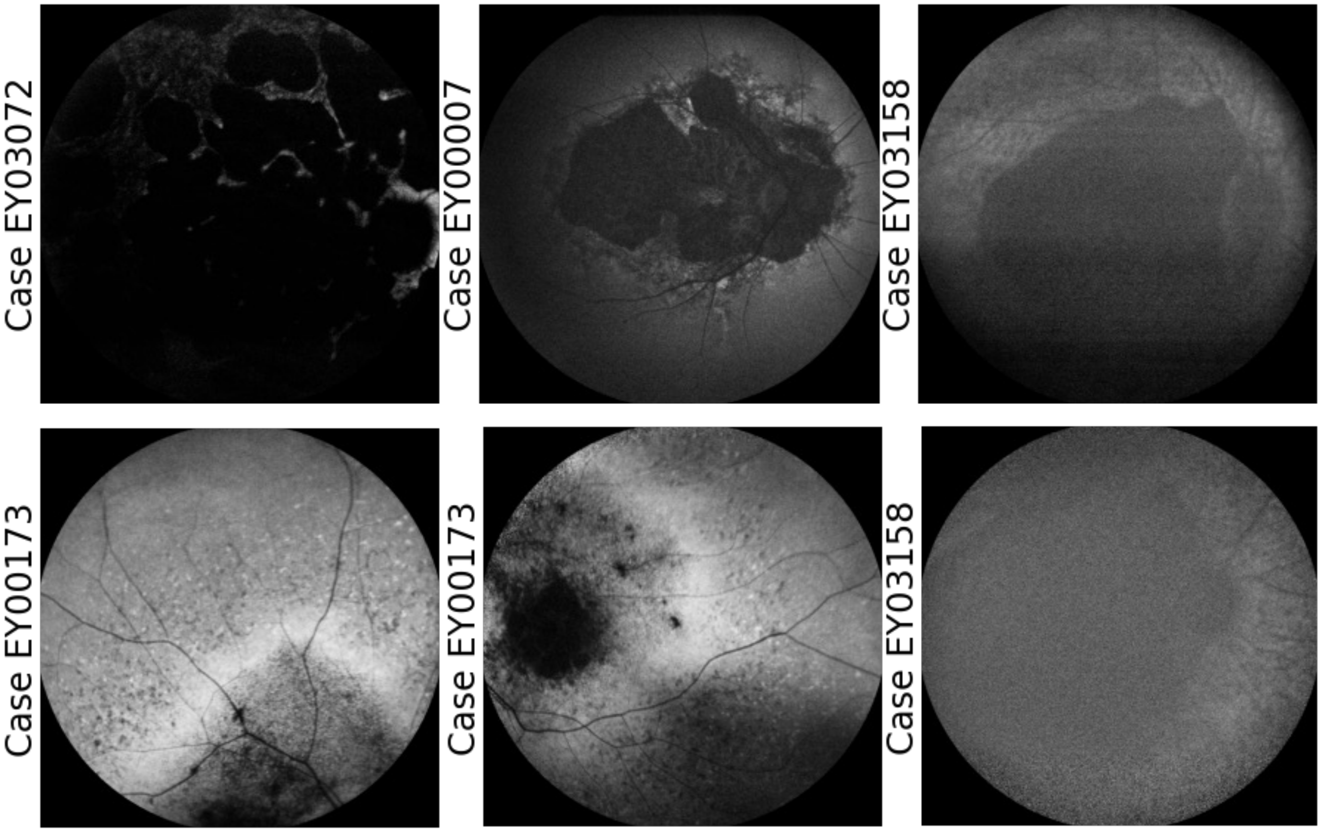

Supplement: Figure S2 [file mmc2.pdf]
