# Supplementary material for: Quantification of Fundus Autofluorescence Features in a Molecularly Characterized Cohort of >3500 Patients with Inherited Retinal Disease from the United Kingdom
Source: Ophthalmol Sci. 2024 Nov 12;5(2):100652. doi: 10.1016/j.xops.2024.100652 (PMC11782848; doi:10.1016/j.xops.2024.100652)
Supplement: Figure S4 [file mmc3.pdf]

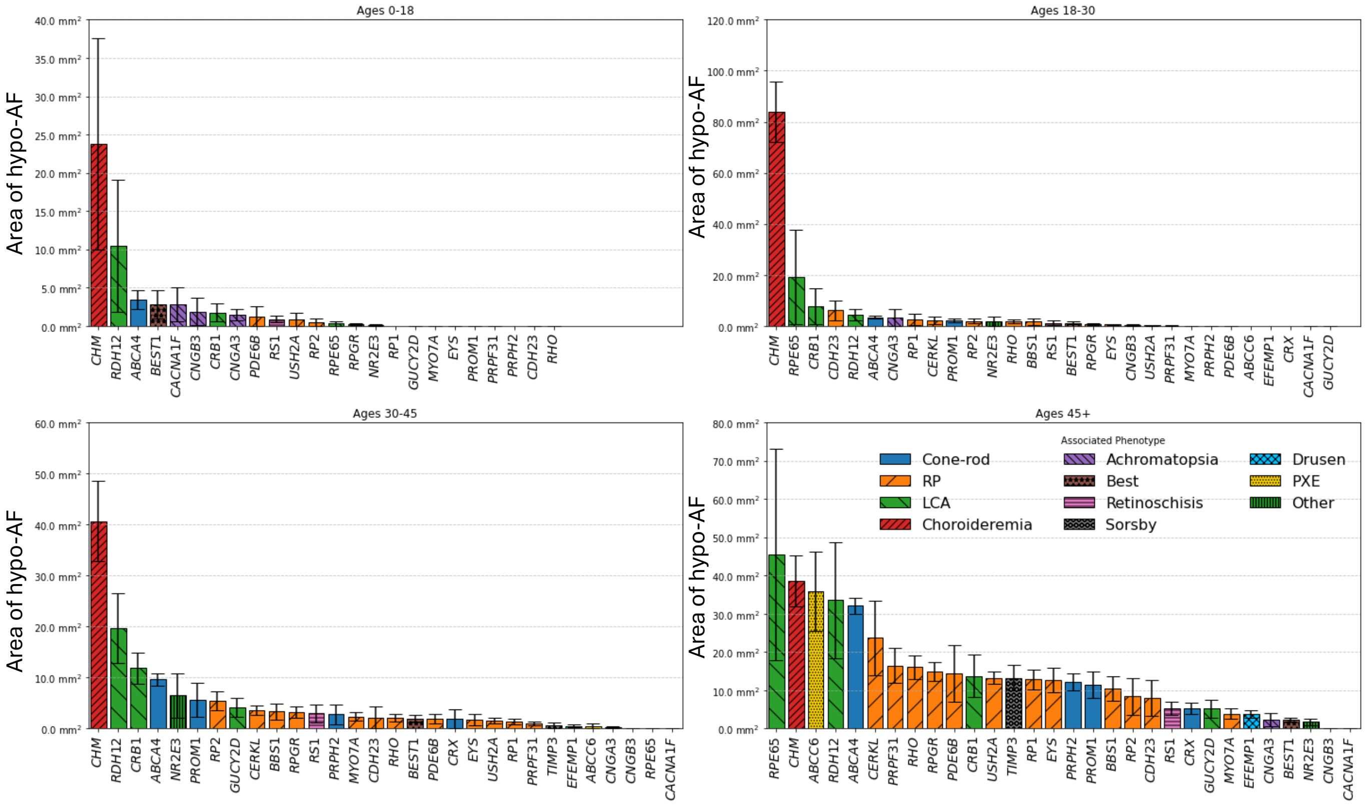

Figure S4: Mean hypo-AF area at presentation by age group (<18, 18-30, 30-45, 45+) across the 30 most common genes (*RPE65* included for reference) broken down by age range. Error bars denote standard error. Each patient is assigned to an age group based on their age at first presentation. Genes are grouped into approximate phenotype groupings denoted by bar styling.
