# Supplementary material for: Quantification of Fundus Autofluorescence Features in a Molecularly Characterized Cohort of >3500 Patients with Inherited Retinal Disease from the United Kingdom
Source: Ophthalmol Sci. 2024 Nov 12;5(2):100652. doi: 10.1016/j.xops.2024.100652 (PMC11782848; doi:10.1016/j.xops.2024.100652)
Supplement: Figure S7 [file mmc6.pdf]

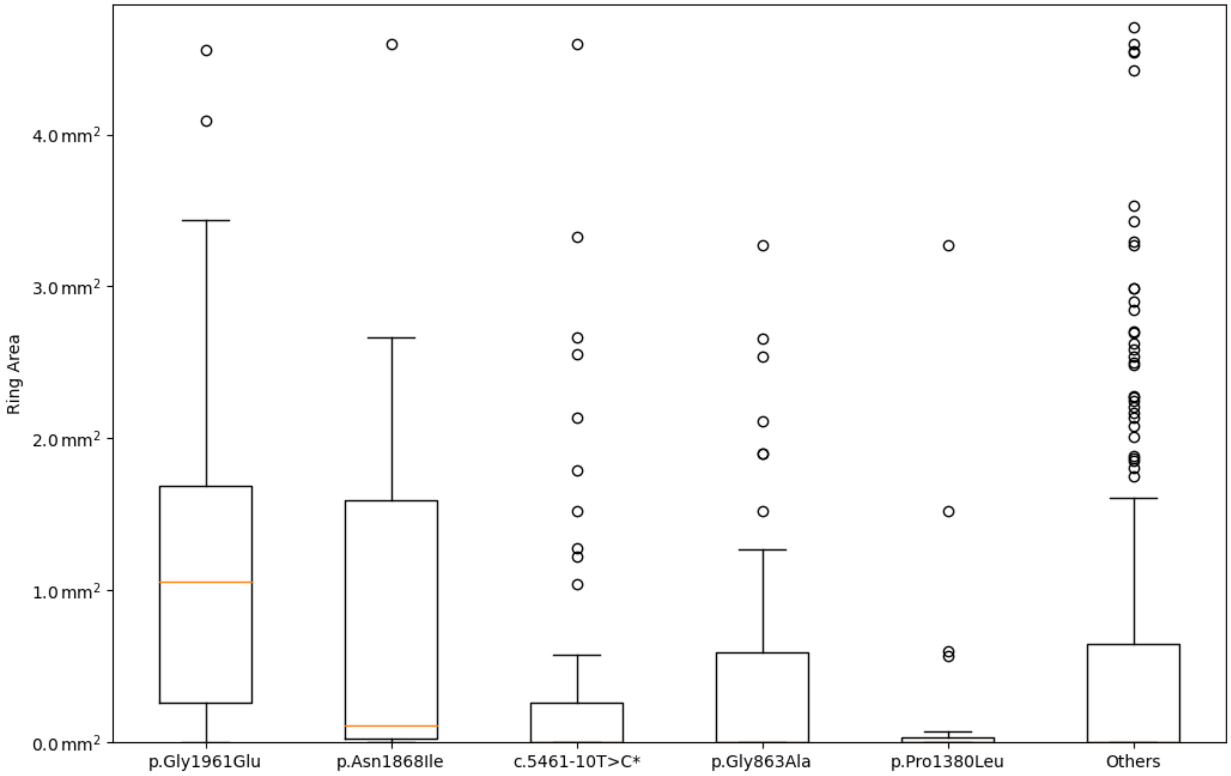

Figure S7: Comparison of the mean per-patient extent of macular ring present for patients with different variants (i.e. patients with at least one copy of the given variant) in *ABCA4*. Axes are truncated to exclude 99th percentile outliers. Most variants of *ABCA4* are not associated with a macular ring of raised AF, apart from p.(Gly1961Glu) which we see reflected in the different distributions of ring area in our data.
