# Supplementary material for: Quantification of Fundus Autofluorescence Features in a Molecularly Characterized Cohort of >3500 Patients with Inherited Retinal Disease from the United Kingdom
Source: Ophthalmol Sci. 2024 Nov 12;5(2):100652. doi: 10.1016/j.xops.2024.100652 (PMC11782848; doi:10.1016/j.xops.2024.100652)
Supplement: Figure S8 [file mmc7.pdf]

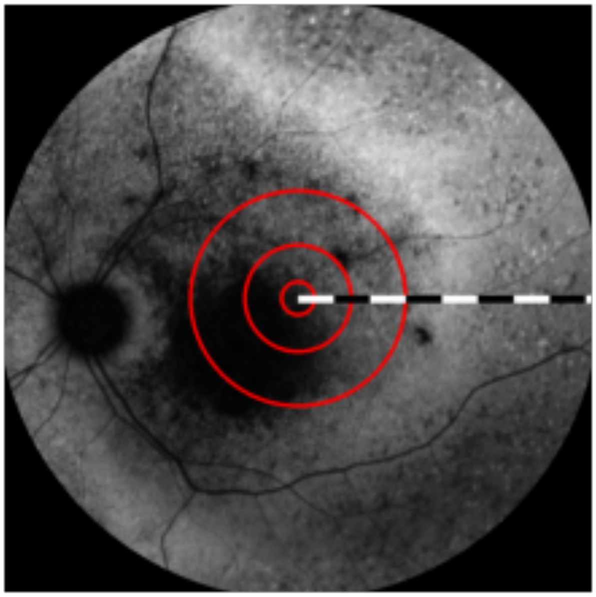

Figure S8: 55-degree FAF image with 0.5mm, 1.5mm, and 3mm radial distances shown (corresponding to 1mm, 3mm, and 6mm diameter ETDRS regions), and scale bar with 1mm gradations.
