# Supplementary material for: Quantification of Fundus Autofluorescence Features in a Molecularly Characterized Cohort of >3500 Patients with Inherited Retinal Disease from the United Kingdom
Source: Ophthalmol Sci. 2024 Nov 12;5(2):100652. doi: 10.1016/j.xops.2024.100652 (PMC11782848; doi:10.1016/j.xops.2024.100652)
Supplement: Figure S11 [file mmc8.pdf]

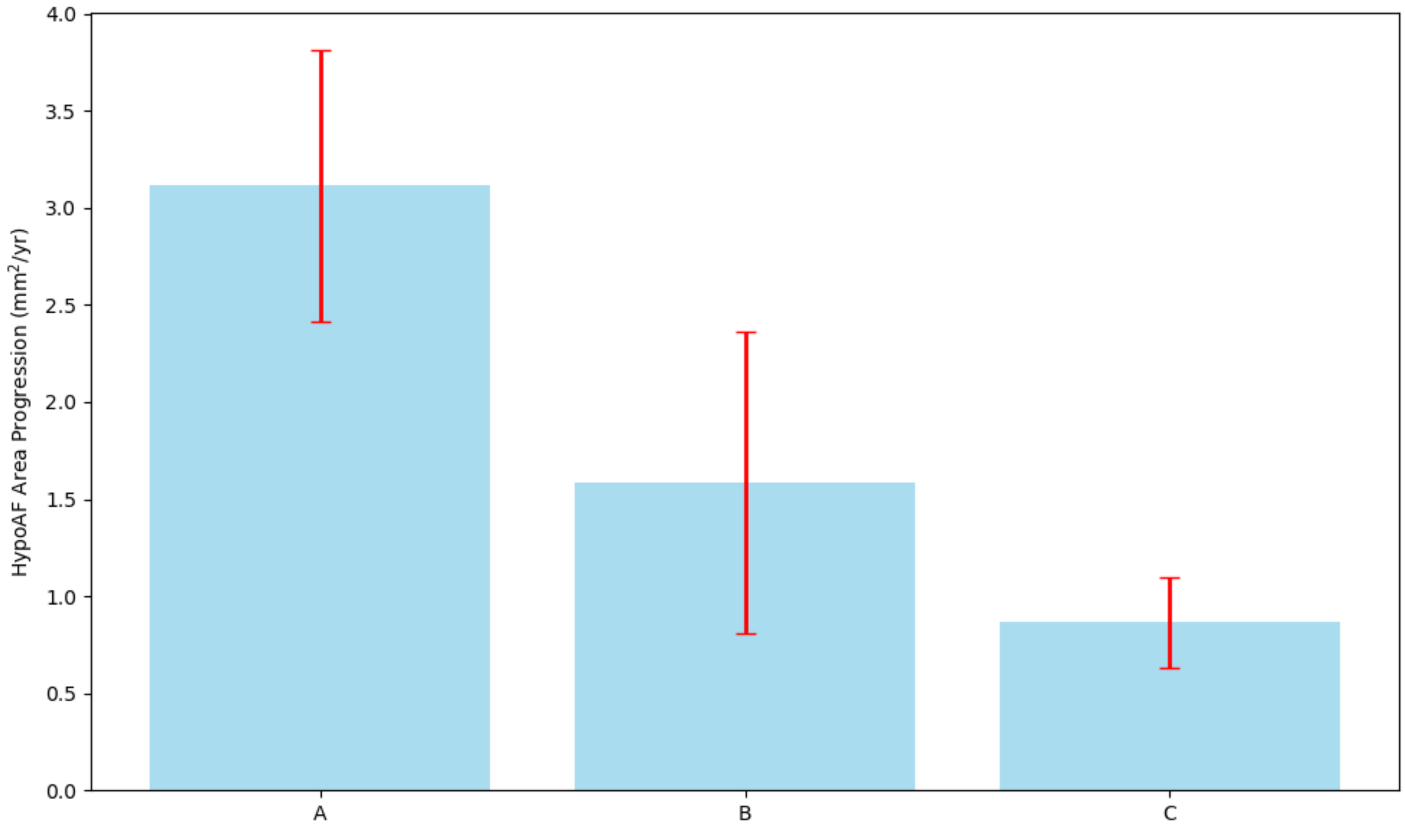

Figure S11: Rate of progression of hypo-AF in mm^2 per year for patients in the three severity classification groups of *ABCA4*. Note that Group A has a higher mean rate of progression than groups B and C, as it corresponds to the group with the highest severity. Error bars denote standard error.
