# Supplementary material for: Quantification of Fundus Autofluorescence Features in a Molecularly Characterized Cohort of >3500 Patients with Inherited Retinal Disease from the United Kingdom
Source: Ophthalmol Sci. 2024 Nov 12;5(2):100652. doi: 10.1016/j.xops.2024.100652 (PMC11782848; doi:10.1016/j.xops.2024.100652)
Supplement: Figure S12 [file mmc9.pdf]

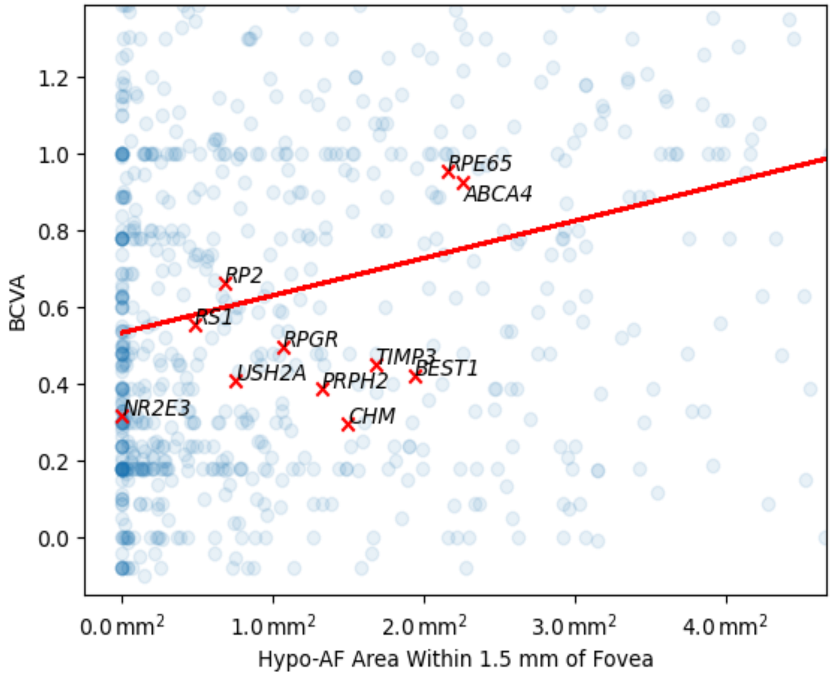

Figure S12: hypo-AF area within 1.5mm of the fovea compared to LogMAR best corrected visual acuity (BCVA) where higher values corresponds to poorer acuity. Axes rescaled to 90th pct of data for legibility. Each circle represents a single patient with mean value across images. Least-squares regression line in red (β=0.083, $p<0.001$). Mean values for select genes are indicated by red crosses. Comparing hypo-AF area within 1.5mm of the fovea and LogMAR best corrected visual acuity (BCVA) showed a positive statistical association (β=0.083, $p<0.001$). However, some genes demonstrated a different relationship from the main trend. For example, in *ABCA4* a worse BCVA was observed than might be expected from hypo-AF coverage, likely because *ABCA4*-associated retinopathy usually initially affects the fovea/central macula. By contrast, *CHM* typically exhibits a spared foveal island despite having significant areas of atrophy, thus accounting for the relatively preserved BCVA.
