# Supplementary material for: Quantification of Fundus Autofluorescence Features in a Molecularly Characterized Cohort of >3500 Patients with Inherited Retinal Disease from the United Kingdom
Source: Ophthalmol Sci. 2024 Nov 12;5(2):100652. doi: 10.1016/j.xops.2024.100652 (PMC11782848; doi:10.1016/j.xops.2024.100652)
Supplement: Figure S13 [file mmc10.pdf]

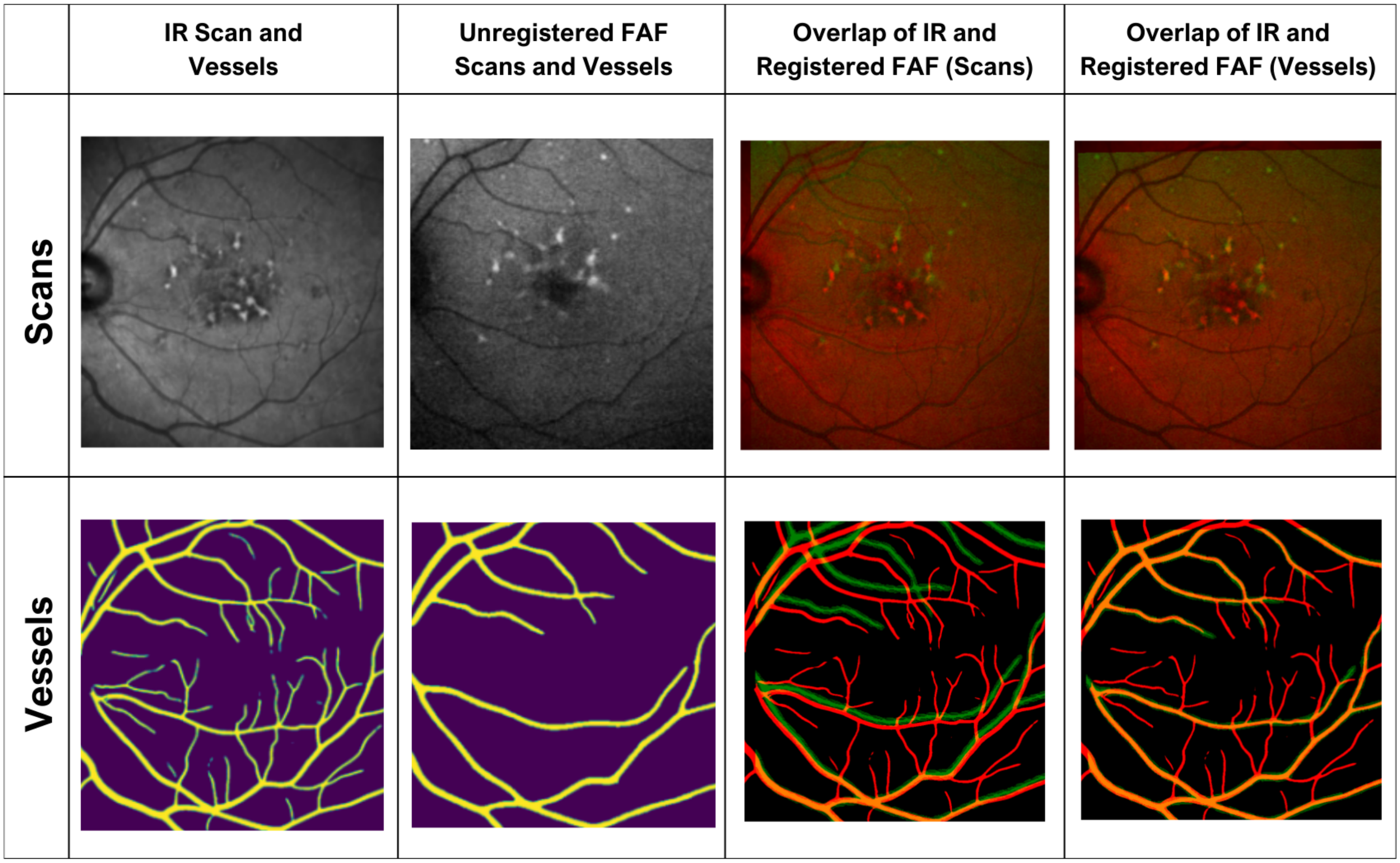

Figure S13: Example showing how vessel tree segmentation improves cross-modality image registration. First row shows the individual and overlaid images, and second row shows corresponding segmented vessel masks. For the overlaid images, the IR image is rendered in red, while the FAF image is rendered in green, enabling overlap to be assessed by looking at the correspondence between the two-colour channels. Vessel trees were extracted using AIRDetect for both the IR and the FAF image. A 30-degree centre-crop of the FAF image was taken to correspond with the IR image, which are typically captured at 30 degrees. Alternatively 30-degree FAF imaging can be used. Results of automatic registration directly on the raw images (scans column) and registration on the vessel trees (vessels column) are shown. In both cases this registration was performed using the SimpleElastix package. As shown by the final column, registering using vessel trees results in better overlap than registering using images alone.
