# Supplementary material for: Quantification of Fundus Autofluorescence Features in a Molecularly Characterized Cohort of >3500 Patients with Inherited Retinal Disease from the United Kingdom
Source: Ophthalmol Sci. 2024 Nov 12;5(2):100652. doi: 10.1016/j.xops.2024.100652 (PMC11782848; doi:10.1016/j.xops.2024.100652)
Supplement: Table S2 [file mmc11.pdf]

Table S2: Overview of annotated dataset for the manually segmented features, considering each feature individually. Not all features were gradable within all images, with some images only annotated for some features. Images for vessel annotations were selected by clinicians and were all gradable. Incidence includes ungradable

**Feature**	**Graded**	**Double graded**	**Partially Gradable**	**Un-Gradable**	**Num Patients**	**Num Genes**	**Present**	**Incidence**
disc	736	207	74	32	573	63	716	97.3%
hypo-AF	736	204	75	32	573	63	482	65.5%
hyper-AF	730	191	77	32	570	63	106	14.5%
ring	729	195	76	32	571	63	212	29.1%
vessels	206	13	*n/a*	*n/a*	127	33	206	100%
