# Supplementary material for: Quantification of Fundus Autofluorescence Features in a Molecularly Characterized Cohort of >3500 Patients with Inherited Retinal Disease from the United Kingdom
Source: Ophthalmol Sci. 2024 Nov 12;5(2):100652. doi: 10.1016/j.xops.2024.100652 (PMC11782848; doi:10.1016/j.xops.2024.100652)
Supplement: Table S3 [file mmc12.pdf]

Table S3: Vessel metrics and their description.

**Vessel Metric**	**Description**
Fractal Dimension	Method that represents geometric complexity of the vascular branching pattern observed in the retina. Essentially how close are the vessels to being "space-filling".
Vessel Density	Ratio between area of vessels and total image area.
Average Width	Average width of vessels.
Distance Tortuosity	Distance tortuosity is a measure of the tortuosity of a path based on the ratio of the actual path length to the straight-line distance between the start and end points of the path[1]
Squared Curvature Tortuosity	Squared curvature tortuosity is a more sophisticated measure of tortuosity that takes into account the curvature along the path, providing a detailed view of its winding nature[1]
Tortuosity Density	Assesses vessel tortuosity by aggregating local contributions, examining the degree to which each turn curve deviates from a smooth curve [2]

.

1. Hart WE, Goldbaum M, Côté B, et al. Measurement and classification of retinal vascular tortuosity. Int J Med Inform 1999;53:239–252.

2. Grisan E, Foracchia M, Ruggeri A. A novel method for the automatic grading of retinal vessel tortuosity. IEEE Trans Med Imaging 2008;27:310–319.
