# Supplementary material for: Quantification of Fundus Autofluorescence Features in a Molecularly Characterized Cohort of >3500 Patients with Inherited Retinal Disease from the United Kingdom
Source: Ophthalmol Sci. 2024 Nov 12;5(2):100652. doi: 10.1016/j.xops.2024.100652 (PMC11782848; doi:10.1016/j.xops.2024.100652)
Supplement: Table S4 [file mmc13.pdf]

Table S4: Feature statistics for all genes. Phenotypes: pheno = most common phenotype presentation according to literature. ACHM = achromatopsia, ALB = albinism, BEST = best disease, CD = cone-dystrophy, CHM = choroidemia, CR = cone-rod, CSNB = congenital stationary night blindness, DR = diabetic retinopathy, FEVR = Familial exudative vitreoretinopathy, GA = Gyrate atrophy, LCA = Leber's congenital amaurosis, MAC = Microphthalmia, anophthalmia, coloboma, MD = macular dystrophy, OA = optic atrophy, PD = pattern dystrophy, PXE = pseudoxanthoma elasticum, RP = retinitis pigmentosa. pat = number of patients. img = number of FAF images. Feature metrics are averaged across all images per gene. Features: % = average incidence in percent, A = averge area in mm2, C = average number of clusters, I = average pixel intensity in percentage, % <6mm = average incidence within 6mm area in percent, FD = vessel fractal dimensions, D = average vessel density, W = average vessel width, DTM = distance tortuosity mean, SCTM = squared curvature tortuosity mean, TDM = tortuosity density mean.

						Disc				Hypo-AF					Hyper-AF					Ring					Vessels					
gene	patient count	image count	Patient Ages			area	num clusters	intensity	% <6mm	incidence	area	num clusters	intensity	% <6mm	incidence	area	num clusters	intensity	% <6mm	incidence	area	num clusters	intensity	% <6mm	Fractal Dimension	Density	Width	Distance tortuosity mean	Squared curvature tortuosity mean	Tortuosity density mean
**Total**	**3491**	**32964**	**min**	**median**	**max**	**2.09**	**1.02**	**13.51%**	**6.86%**	**61.15%**	**13.04**	**1.46**	**13.02%**	**48.17%**	**25.11%**	**0.15**	**0.43**	**57.14%**	**52.81%**	**43.71%**	**1.19**	**1.78**	**54.10%**	**77.92%**	**1.25**	**0.06**	**186.52**	**6.45**	**98.03**	**0.70**
ABCA4	873	7926	9	46	93	2.19	1.01	9.25%	13.74%	80.29%	19.81	1.45	11.13%	61.34%	18.04%	0.05	0.33	66.40%	48.28%	33.46%	0.57	1.58	59.62%	81.76%	1.33	0.08	184.81	4.81	61.64	0.71
ABCC6	17	268	26	62	90	1.53	1.03	16.85%	17.58%	69.03%	21.44	2.08	14.31%	32.63%	54.85%	0.23	1.20	62.52%	23.36%	8.21%	0.03	0.15	43.31%	38.00%	1.37	0.08	196.68	6.17	80.72	0.73
ABHD12	4	44	26	33.5	59	2.50	1.00	11.17%	3.83%	77.27%	15.90	2.48	11.45%	49.64%	27.27%	0.02	0.27	35.63%	66.67%	2.27%	4.01E-05	0.00	47.94%	100.00%	1.19	0.06	179.19	5.24	36.36	0.68
ADAMTSL4	1	12	33	33	33	2.27	1.00	11.05%	0.00%	25.00%	0.02	0.33	9.49%	0.00%	0.00%	0.00	0.00			16.67%	0.03	0.17	42.55%	100.00%	1.35	0.07	213.39	4.03	53.32	0.69
ADGRV1	12	102	26	51.5	77	2.54	1.00	17.39%	1.46%	63.73%	4.88	1.19	17.73%	46.66%	8.82%	0.08	0.12	64.25%	86.49%	86.27%	1.09	3.35	52.19%	99.03%	1.13	0.02	182.36	8.66	140.31	0.67
AGBL5	1	10	73	73	73	0.54	1.60	9.81%	0.00%	100.00%	9.49	4.80	2.21%	5.33%	0.00%	0.00	0.00			0.00%	0.00	0.00			1.31	0.04	206.99	8.55	91.48	0.72
AHI1	6	49	17	52	65	2.85	1.00	10.99%	5.88%	65.31%	12.24	0.86	12.82%	53.64%	10.20%	0.01	0.06	42.02%	60.00%	42.86%	0.50	0.96	54.65%	85.91%	1.14	0.03	180.38	5.46	58.98	0.70
AIPL1	2	25	9	15	56	2.48	1.00	9.60%	0.06%	8.00%	0.11	0.40	2.85%	2.66%	32.00%	0.03	0.24	47.47%	0.00%	80.00%	8.66	2.12	40.42%	41.64%	1.12	0.04	229.54	5.70	40.85	0.71
ALMS1	1	11	15	28	48	2.36	1.00	13.89%	0.00%	0.00%	0.00	0.00			0.00%	0.00	0.00			0.00%	0.00	0.00			1.10	0.04	186.00	5.01	67.32	0.70
AMACR	2	12	61	62.5	64	1.69	1.00	18.99%	0.00%	41.67%	0.10	0.42	13.05%	77.46%	66.67%	0.10	0.67	56.90%	3.57%	25.00%	0.09	0.33	43.51%	75.00%	1.15	0.04	191.50	7.62	110.12	0.65
ARHGEF18	3	39	44	45	60	2.34	1.00	15.63%	1.72%	46.15%	2.88	1.13	11.33%	6.43%	33.33%	0.12	0.69	41.63%	58.53%	56.41%	0.99	2.08	48.92%	48.09%	1.22	0.05	201.54	8.35	48.53	0.64
ARL3	1	10	29	29	29	3.00	1.00	7.28%	0.00%	40.00%	0.00	0.20	7.68%	0.00%	0.00%	0.00	0.00			0.00%	0.00	0.00			1.45	0.12	198.62	4.48	27.12	0.74
ARL6	2	14	43	43.5	47	1.93	1.00	21.11%	22.91%	92.86%	35.47	1.43	16.59%	46.02%	50.00%	0.23	0.57	43.28%	85.71%	0.00%	0.00	0.00			0.89	0.00	123.00	25.43	1151.60	0.55
ATF6	2	15	30	31.5	33	2.00	1.00	17.23%	0.55%	53.33%	2.88	0.53	13.46%	81.15%	46.67%	0.03	0.53	55.29%	38.45%	13.33%	0.02	0.13	29.13%	100.00%	1.28	0.06	229.27	4.11	47.41	0.75
ATXN7	1	9	77	77	77	1.44	1.00	18.74%	0.00%	22.22%	0.14	0.78	0.50%	100.00%	55.56%	0.05	1.22	64.87%	0.00%	0.00%	0.00	0.00			1.36	0.08	243.18	10.45	149.14	0.73
BBS1	31	286	16	38	78	2.59	1.02	10.41%	7.52%	67.48%	8.35	1.29	11.91%	56.94%	25.87%	0.15	0.38	47.26%	86.85%	32.52%	0.84	1.03	56.50%	72.71%	1.18	0.05	186.86	9.66	214.92	0.70
BBS10	4	35	17	36	39	2.47	1.09	12.38%	30.85%	91.43%	8.64	1.37	11.18%	41.65%	0.00%	0.00	0.00			0.00%	0.00	0.00			0.96	0.01	172.29	13.56	349.29	0.68
BBS12	2	22	21	35	60	2.19	1.05	13.20%	16.11%	95.45%	22.14	2.45	7.56%	44.37%	27.27%	0.05	0.36	57.05%	57.80%	45.45%	0.34	0.91	42.80%	55.42%	1.22	0.04	193.66	8.94	93.01	0.74
BBS2	2	14	36	40	44	2.96	1.00	7.20%	19.31%	100.00%	4.91	1.50	14.15%	76.73%	0.00%	0.00	0.00			57.14%	1.85	1.43	63.87%	89.38%	1.29	0.04	134.42	6.12	201.98	0.74
BBS5	2	19	32	53	74	3.50	1.00	12.69%	3.59%	5.26%	0.00	0.05	23.42%	0.00%	31.58%	0.08	0.53	46.02%	82.37%	84.21%	0.93	3.95	47.36%	80.33%	1.38	0.08	206.60	11.55	199.06	0.76
BEST1	133	1461	8	46	86	1.84	1.01	11.59%	4.14%	40.45%	2.58	0.90	11.50%	59.70%	57.49%	0.73	1.09	61.18%	59.81%	57.49%	1.28	1.83	56.31%	77.22%	1.38	0.10	203.18	4.46	49.43	0.71
C1QTNF5	10	73	50	73.5	87	1.85	1.00	13.85%	3.18%	78.08%	27.18	1.93	15.97%	23.12%	8.22%	0.01	0.07	59.98%	50.00%	1.37%	0.02	0.07	51.84%	100.00%	1.35	0.08	199.82	4.78	43.26	0.73
C21ORF2	5	44	27	37.5	57	2.56	1.00	11.52%	0.37%	20.45%	0.76	0.25	32.97%	17.62%	0.00%	0.00	0.00			52.27%	1.00	1.23	56.98%	45.38%	1.28	0.07	191.13	4.31	52.74	0.73
C2ORF71	10	85	52	52	52	2.38	1.01	13.39%	6.76%	96.47%	19.08	1.75	12.65%	65.48%	7.06%	0.00	0.07	55.40%	83.33%	23.53%	0.63	0.86	62.86%	62.54%	1.05	0.03	177.08	13.59	252.66	0.63
CABP4	4	29	9	39	48	1.33	1.03	14.68%	3.45%	20.69%	0.01	0.79	0.03%	33.33%	17.24%	0.00	0.21	53.22%	0.00%	0.00%	0.00	0.00			1.11	0.03	224.24	2.22	20.98	0.71
CACNA1F	30	265	4	18	79	2.04	1.01	14.76%	0.01%	5.66%	0.91	0.20	7.13%	42.39%	12.83%	0.01	0.15	59.85%	2.47%	3.77%	0.01	0.06	53.57%	79.82%	1.21	0.05	218.03	9.24	83.77	0.73
CACNA2D4	1	16	40	40	40	1.06	1.00	17.29%	0.00%	100.00%	0.09	4.06	0.11%	80.36%	0.00%	0.00	0.00			0.00%	0.00	0.00			1.49	0.10	210.24	3.97	30.23	0.69
CDH23	17	260	15	40	74	2.05	1.01	14.31%	6.45%	16.15%	0.71	0.47	10.44%	41.97%	19.62%	0.23	0.50	57.64%	34.17%	77.31%	2.46	2.73	47.34%	67.30%	1.05	0.02	198.91	7.97	177.84	0.68
CDH3	3	21	32	38	63	1.65	1.19	15.05%	0.00%	100.00%	25.42	2.24	11.58%	48.30%	42.86%	0.82	1.43	45.35%	0.00%	28.57%	0.11	1.19	43.43%	0.00%	1.27	0.05	219.90	3.19	17.80	0.63
CDHR1	18	144	26	41	82	2.48	1.04	12.55%	5.10%	80.56%	7.39	1.71	11.82%	66.14%	22.92%	0.18	0.26	49.52%	75.30%	36.81%	1.11	1.25	53.78%	70.56%	1.22	0.04	180.64	7.80	77.75	0.68
CEP290	16	166	6	33	57	2.22	0.99	14.25%	3.10%	19.88%	0.90	0.69	6.43%	48.94%	13.25%	0.10	0.25	62.19%	13.64%	70.48%	1.40	2.10	54.86%	80.08%	1.16	0.04	186.46	10.90	281.31	0.71
CEP78	1	5	72	72	72	1.47	1.00	20.86%	12.22%	100.00%	37.38	3.80	22.08%	9.46%	100.00%	0.56	1.80	53.42%	18.37%	100.00%	3.94	2.00	51.21%	87.14%	1.01	0.01	213.43	3.91	40.37	0.77
CERKL	22	249	20	40	80	2.63	1.00	12.26%	9.15%	82.33%	10.33	1.43	12.08%	51.71%	17.67%	0.02	0.21	46.03%	88.64%	29.72%	1.04	0.95	58.07%	65.30%	1.16	0.04	179.55	7.20	95.84	0.67
CHM	109	1731	11	42	86	1.45	1.06	20.61%	2.84%	82.61%	51.77	3.28	19.89%	20.95%	48.76%	0.26	0.83	54.55%	37.86%	5.55%	0.03	0.08	43.19%	32.21%	1.30	0.06	190.34	6.14	104.36	0.69
CLCC1	1	12	25	25	25	2.37	1.00	22.89%	0.00%	25.00%	0.01	0.33	12.78%	0.00%	0.00%	0.00	0.00			33.33%	0.54	0.33	54.51%	100.00%	0.79	0.00	137.72	2.37	1.88	0.29
CLN3	10	147	13	47	62	2.23	1.03	16.06%	8.51%	34.01%	1.75	1.17	13.96%	37.43%	2.72%	0.00	0.06	57.99%	25.00%	47.62%	0.52	1.97	49.10%	92.66%	1.29	0.06	217.27	7.86	143.39	0.73
CLRN1	10	80	22	47.5	75	1.40	1.04	19.90%	4.06%	60.00%	9.38	1.71	15.71%	23.13%	23.75%	0.06	0.41	53.97%	63.16%	51.25%	0.97	1.44	52.49%	94.12%	1.02	0.01	143.88	15.66	221.72	0.67
CNGA1	8	54	34	53	90	2.39	1.00	18.34%	2.16%	16.67%	2.91	0.20	20.70%	75.96%	3.70%	0.22	0.15	59.32%	21.66%	22.22%	1.32	0.89	60.53%	65.29%	1.30	0.07	206.51	7.38	89.46	0.70
CNGA3	36	289	4	23	67	1.74	1.01	13.81%	14.49%	31.14%	1.48	0.96	7.06%	56.25%	7.96%	0.05	0.17	53.96%	31.76%	35.64%	0.32	0.75	50.03%	74.89%	1.24	0.05	209.19	5.17	70.94	0.70
CNGB1	28	198	26	58	88	1.59	1.03	13.07%	1.74%	79.80%	19.98	2.03	10.08%	25.60%	46.46%	0.57	0.95	54.04%	50.81%	70.71%	3.63	2.64	54.52%	64.45%	1.17	0.03	160.01	7.17	90.08	0.68
CNGB3	39	288	5	29.5	80	1.91	0.99	14.86%	12.72%	12.50%	0.16	0.38	6.73%	73.57%	10.42%	0.01	0.12	52.72%	63.89%	32.64%	0.20	0.63	54.64%	92.23%	1.24	0.06	205.53	6.42	67.09	0.70
COL11A1	1	8	30	42	56	2.64	1.00	6.77%	2.55%	100.00%	0.73	2.75	6.21%	61.71%	37.50%	0.01	0.38	34.48%	33.33%	12.50%	0.04	0.38	40.40%	100.00%	1.13	0.04	178.64	3.60	29.13	0.58
COL18A1	2	2	9	27	42	0.26	1.00	13.17%	0.00%	50.00%	34.50	3.00	19.81%	14.65%	50.00%	0.25	0.50	55.09%	1.23%	0.00%	0.00	0.00			1.01	0.02	205.69	2.51	5.27	0.86
COL2A1	1	8	61	66	71	1.71	1.38	19.62%	13.38%	100.00%	27.44	2.63	8.53%	6.64%	75.00%	0.56	0.88	63.15%	0.00%	25.00%	0.07	0.25	46.61%	27.85%	1.15	0.03	188.28	3.09	21.79	0.69
COL4A1	1	7	48	48	48	1.93	1.00	16.31%	0.00%	0.00%	0.00	0.00			0.00%	0.00	0.00			0.00%	0.00	0.00			1.50	0.14	188.66	3.98	37.88	0.70
COL4A5	1	10	40	40	40	1.61	1.00	12.84%	0.00%	0.00%	0.00	0.00			0.00%	0.00	0.00			100.00%	11.30	4.10	57.72%	80.63%	1.20	0.03	194.98	8.70	85.90	0.81
CRB1	47	455	8	38	80	1.97	1.02	14.70%	3.63%	55.16%	8.30	1.22	15.60%	40.44%	43.96%	0.41	0.89	46.39%	54.00%	43.74%	1.73	1.35	48.91%	59.21%	1.11	0.04	178.82	7.20	83.21	0.69
CRX	26	236	20	61	85	2.11	1.00	15.62%	5.73%	70.34%	5.51	1.39	15.62%	62.69%	16.95%	0.05	0.26	50.74%	29.92%	59.32%	2.23	4.22	52.94%	65.95%	1.33	0.07	192.40	9.58	119.49	0.72
CTNNA1	4	10	33	39	65	1.51	1.30	18.54%	0.00%	40.00%	1.50	2.20	1.60%	13.10%	60.00%	0.23	1.20	56.33%	99.44%	50.00%	0.04	0.80	56.60%	80.51%	1.42	0.09	192.93	4.01	26.94	0.71
CWC27	1	1	19	19	19	0.30	1.00	21.28%	0.00%	0.00%	0.00	0.00			0.00%	0.00	0.00			0.00%	0.00	0.00			0.56	0.00	80.80	3.13	1.54	0.41
CYP4V2	17	106	21	47	86	1.18	1.06	22.53%	3.07%	86.79%	35.05	3.20	16.02%	33.68%	15.09%	0.02	0.20	49.14%	61.88%	3.77%	0.01	0.04	32.83%	50.00%	1.00	0.02	144.85	4.90	28.37	0.63
DRAM2	8	70	43	57	74	2.46	1.00	14.67%	9.78%	90.00%	5.97	1.50	17.68%	89.28%	2.86%	0.00	0.03	29.53%	100.00%	34.29%	0.29	0.96	59.39%	73.82%	1.37	0.08	190.91	5.03	65.16	0.73
DYNC2H1	1	3	51	51	51	3.05	1.00	33.60%	0.00%	33.33%	0.30	0.33	35.71%	100.00%	0.00%	0.00	0.00			66.67%	0.76	1.67	61.57%	100.00%	1.07	0.02	155.01	7.69	157.23	0.49
EFEMP1	30	249	26	58.5	97	1.77	1.00	15.28%	5.98%	78.71%	5.15	1.84	14.24%	82.36%	77.91%	0.24	1.55	61.84%	79.37%	24.10%	0.18	0.66	60.03%	82.55%	1.43	0.10	208.55	4.31	39.45	0.72
EYS	70	628	10	59	88	2.36	1.02	15.16%	3.37%	65.92%	9.55	1.64	13.27%	27.28%	23.41%	0.23	0.40	57.64%	42.90%	72.45%	2.08	2.76	52.59%	76.78%	1.16	0.03	172.31	7.39	108.29	0.69
FAM161A	8	62	27	50	63	2.12	1.05	14.58%	14.56%	66.13%	11.60	2.02	13.90%	40.33%	19.35%	0.27	0.35	46.78%	58.46%	83.87%	1.83	2.77	51.04%	90.53%	1.10	0.02	168.97	5.66	45.07	0.65
FLVCR1	4	27	52	53	59	1.01	0.93	16.45%	3.53%	48.15%	2.58	1.85	5.93%	41.36%	18.52%	0.15	0.37	75.14%	20.00%	74.07%	3.76	1.78	49.80%	78.55%	1.06	0.02	176.65	9.58	88.25	0.70
FZD4	2	9	9	14	67	2.78	1.11	9.17%	16.13%	66.67%	2.59	1.11	9.48%	58.83%	44.44%	0.12	0.56	78.35%	0.00%	33.33%	0.02	0.56	71.29%	0.00%	1.40	0.10	211.97	4.37	35.44	0.66
GNAT2	2	31	30	32	34	2.03	1.00	15.64%	0.00%	0.00%	0.00	0.00			9.68%	0.01	0.10	52.77%	0.00%	0.00%	0.00	0.00			1.35	0.08	230.33	4.50	55.07	0.73

						Disc				Hypo-AF					Hyper-AF					Ring					Vessels					
gene	patient count	image count	Patient Ages			area	num clusters	intensity	% <6mm	incidence	area	num clusters	intensity	% <6mm	incidence	area	num clusters	intensity	% <6mm	incidence	area	num clusters	intensity	% <6mm	Fractal Dimension	Density	Width	Distance tortuosity mean	Squared curvature tortuosity mean	Tortuosity density mean
**Total**	**3491**	**32964**	**min**	**median**	**max**	**2.09**	**1.02**	**13.51%**	**6.86%**	**61.15%**	**13.04**	**1.46**	**13.02%**	**48.17%**	**25.11%**	**0.15**	**0.43**	**57.14%**	**52.81%**	**43.71%**	**1.19**	**1.78**	**54.10%**	**77.92%**	**1.25**	**0.06**	**186.52**	**6.45**	**98.03**	**0.70**
GPR143	6	28	14	35	69	2.31	1.00	13.46%	0.74%	0.00%	0.00	0.00			0.00%	0.00	0.00			0.00%	0.00	0.00			1.43	0.11	209.76	5.46	61.22	0.71
GPR179	2	12	13	25.5	40	2.71	1.00	13.40%	3.57%	50.00%	0.95	0.67	21.58%	100.00%	0.00%	0.00	0.00			50.00%	1.97	1.33	51.76%	100.00%	1.30	0.06	221.14	9.26	157.48	0.75
GRK1	1	4	48	48	48	3.41	1.00	22.44%	0.00%	100.00%	2.02	1.00	31.29%	97.92%	0.00%	0.00	0.00			100.00%	1.88	2.25	54.96%	100.00%	1.28	0.04	185.11	8.14	150.90	0.76
GRM6	5	17	9	27	59	1.69	1.12	14.61%	0.00%	41.18%	0.42	0.82	8.63%	9.56%	23.53%	0.04	0.24	60.87%	0.00%	11.76%	0.02	0.18	73.19%	0.00%	1.19	0.06	162.84	3.47	23.22	0.66
GUCA1A	11	79	29	58	73	2.25	1.01	16.55%	9.69%	41.77%	2.57	0.80	17.77%	58.47%	25.32%	0.02	0.24	57.05%	65.00%	69.62%	1.14	1.89	60.80%	93.77%	1.21	0.07	175.35	4.35	34.07	0.61
GUCY2D	28	256	13	45	74	2.00	1.00	14.65%	6.51%	48.05%	5.25	0.86	17.50%	71.23%	17.58%	0.22	0.30	61.24%	35.99%	60.94%	1.51	2.94	53.11%	74.98%	1.29	0.07	197.87	5.41	64.41	0.72
HGSNAT	11	150	53	69	86	2.04	1.01	19.73%	7.15%	87.33%	25.58	2.53	19.35%	23.44%	47.33%	0.61	0.89	59.42%	60.81%	76.00%	3.46	2.91	50.43%	84.38%	1.16	0.04	173.35	6.01	52.57	0.68
HPS6	1	2	7	10	57	1.98	1.00	10.33%	0.00%	0.00%	0.00	0.00			50.00%	0.01	0.50	60.38%	0.00%	100.00%	0.66	1.50	67.26%	91.78%	1.43	0.12	224.18	5.33	53.82	0.70
IFT140	8	74	0	46.5	95	2.45	1.01	13.92%	2.45%	72.97%	26.72	1.93	14.83%	41.25%	28.38%	0.04	0.34	51.37%	54.63%	27.03%	0.57	0.77	47.30%	63.40%	1.05	0.03	178.13	8.60	175.65	0.64
IMPDH1	6	102	18	60	85	1.03	1.15	20.24%	1.41%	16.67%	0.74	0.49	7.60%	6.21%	70.59%	0.69	1.00	78.22%	1.37%	74.51%	5.84	1.47	43.96%	42.58%	1.10	0.03	186.45	11.70	355.53	0.72
IMPG1	2	10	45	66	88	2.57	1.00	13.09%	10.11%	70.00%	0.63	0.60	10.29%	100.00%	30.00%	0.16	0.30	62.18%	66.67%	70.00%	0.25	1.60	61.95%	53.47%	1.41	0.10	206.75	9.02	147.19	0.72
IMPG2	12	101	26	40	75	2.61	1.02	14.77%	3.69%	58.42%	4.78	1.06	13.69%	30.79%	9.90%	0.05	0.21	57.06%	57.45%	27.72%	0.26	0.71	44.02%	68.09%	1.13	0.03	197.91	7.92	135.19	0.68
INPP5E	4	27	28	33.5	47	1.88	1.04	9.15%	3.72%	37.04%	0.95	1.85	1.51%	35.60%	7.41%	0.00	0.07	42.62%	100.00%	92.59%	3.00	4.00	58.85%	80.90%	1.38	0.07	197.45	4.24	56.23	0.68
IQCB1	8	48	13	45	80	2.33	1.00	10.80%	18.66%	18.75%	1.36	0.27	14.41%	34.34%	14.58%	0.10	0.19	41.52%	51.81%	85.42%	3.57	3.42	38.49%	71.76%	1.19	0.04	214.53	4.42	35.61	0.71
JAG1	1	10	36	36	36	2.85	1.00	12.21%	52.78%	0.00%	0.00	0.00			0.00%	0.00	0.00			0.00%	0.00	0.00			1.41	0.09	195.27	6.02	179.10	0.77
KCNJ13	1	1	44	44	44	0.04	1.00	6.74%	0.00%	100.00%	24.14	1.00	7.38%	51.12%	0.00%	0.00	0.00			0.00%	0.00	0.00			0.78	0.00	132.76	11.98	34.13	0.51
KCNV2	24	159	9	35	77	2.06	1.01	12.71%	5.01%	35.85%	1.77	0.75	13.62%	84.52%	9.43%	0.01	0.08	53.98%	67.72%	52.20%	0.92	2.87	51.85%	82.53%	1.27	0.07	199.15	6.01	79.68	0.73
KIF11	1	1	10	14	18	1.55	1.00	16.73%	0.00%	0.00%	0.00	0.00			0.00%	0.00	0.00			0.00%	0.00	0.00			0.83	0.00	158.06	1.13	1.25	0.67
KIZ	1	2	22	22	22	2.77	1.00	8.86%	0.00%	0.00%	0.00	0.00			0.00%	0.00	0.00			100.00%	2.97	9.50	46.16%	100.00%	1.41	0.10	108.94	8.33	73.84	0.77
KLHL7	8	75	31	44	80	2.40	0.99	9.63%	0.22%	70.67%	13.00	1.92	13.58%	30.31%	24.00%	0.29	0.32	55.77%	93.11%	84.00%	3.32	5.05	49.44%	89.12%	1.08	0.02	155.27	4.88	46.52	0.68
LAMA1	3	64	39	42	44	1.82	1.03	15.16%	11.46%	73.44%	14.25	1.59	11.84%	3.88%	62.50%	0.68	1.02	51.90%	29.27%	68.75%	1.70	3.20	49.59%	34.35%	1.28	0.05	230.37	2.95	26.37	0.79
LCA3	1	2	29	29	29	0.21	1.00	8.14%	0.00%	0.00%	0.00	0.00			0.00%	0.00	0.00			100.00%	1.05	4.00	23.52%	90.24%	0.89	0.00	173.64	2.87	41.26	0.69
LCA5	3	22	11	31	41	1.68	0.95	12.37%	1.64%	77.27%	21.91	0.95	12.25%	36.27%	0.00%	0.00	0.00			0.00%	0.00	0.00			0.91	0.01	163.08	2.27	11.30	0.61
LHON	3	9	10	56	77	1.93	1.00	16.85%	11.09%	22.22%	0.26	0.44	13.18%	0.00%	22.22%	0.01	0.22	30.78%	0.00%	22.22%	0.05	0.44	30.19%	0.00%	1.29	0.07	200.32	11.21	156.87	0.75
LRAT	1	6	45	56.5	68	1.27	1.00	47.65%	0.00%	0.00%	0.00	0.00			83.33%	0.18	1.00	65.92%	38.33%	50.00%	0.10	0.50	67.11%	67.84%	0.93	0.01	198.61	64.14	3810.75	0.84
MAK	1	6	60	60	60	1.97	1.00	22.73%	0.00%	100.00%	2.96	3.17	15.83%	19.65%	0.00%	0.00	0.00			50.00%	0.48	2.17	47.06%	97.53%	1.12	0.01	163.64	8.17	284.51	0.70
MERTK	16	199	17	29	62	2.32	1.01	16.10%	2.09%	50.25%	4.65	0.78	13.72%	67.63%	40.70%	0.09	0.58	50.57%	74.20%	49.25%	0.26	1.11	52.78%	86.12%	1.12	0.03	203.52	7.47	143.86	0.70
MFRP	5	95	11	36.5	57	1.52	1.05	9.39%	16.23%	63.16%	4.52	1.24	5.81%	12.76%	49.47%	0.14	0.84	66.66%	11.94%	10.53%	0.11	0.38	35.89%	90.00%	1.45	0.12	219.12	4.58	59.51	0.72
MFSD8	12	132	35	52	86	2.50	1.02	16.29%	11.16%	90.91%	9.35	1.48	16.31%	77.20%	4.55%	0.00	0.06	53.64%	83.33%	29.55%	0.71	1.06	53.56%	79.47%	1.16	0.05	180.60	4.76	37.14	0.68
MKKS	2	41	21	23	25	2.79	1.07	10.91%	23.12%	73.17%	0.82	1.10	13.35%	61.34%	58.54%	0.13	0.59	41.18%	57.57%	0.00%	0.00	0.00			0.99	0.01	199.05	17.37	849.58	0.71
MT-ATP6	2	30	39	49	59	1.87	1.00	8.18%	0.02%	100.00%	14.49	2.27	11.39%	38.65%	76.67%	0.64	1.57	38.90%	71.42%	50.00%	0.25	0.57	37.80%	83.24%	0.90	0.01	142.39	15.17	584.20	0.73
MT-ND1, MT-N	2	11	25	51	82	1.94	1.00	22.93%	0.00%	0.00%	0.00	0.00			0.00%	0.00	0.00			0.00%	0.00	0.00			1.40	0.10	204.03	6.87	77.56	0.74
MT-TL1	18	121	34	61	82	2.45	1.00	15.52%	9.29%	99.17%	27.19	1.83	17.96%	60.58%	8.26%	0.01	0.09	72.61%	53.87%	2.48%	0.03	0.05	57.33%	41.27%	1.38	0.08	200.53	4.72	56.76	0.73
MYO7A	49	489	12	45	85	2.07	0.99	15.77%	2.33%	42.33%	2.37	0.95	18.00%	32.93%	28.02%	0.41	0.97	53.69%	21.82%	73.62%	3.24	3.26	47.00%	57.83%	1.09	0.03	182.09	11.00	296.77	0.70
NDP	1	4	37	37.5	38	2.60	1.00	6.09%	25.00%	50.00%	10.17	2.50	6.10%	0.00%	50.00%	0.05	0.50	68.53%	50.00%	0.00%	0.00	0.00			1.31	0.09	193.26	4.32	60.09	0.72
NEK1	1	2	29	29	29	2.26	1.00	9.98%	0.00%	0.00%	0.00	0.00			50.00%	0.01	0.50	50.11%	0.00%	0.00%	0.00	0.00			1.44	0.11	213.59	2.76	17.96	0.72
NHS	1	5	49	49	49	2.52	1.00	13.94%	2.33%	0.00%	0.00	0.00			0.00%	0.00	0.00			0.00%	0.00	0.00			1.39	0.09	214.62	5.06	34.29	0.75
NMNAT1	1	6	12	21	66	2.15	1.00	22.95%	0.00%	100.00%	6.93	2.33	25.50%	11.76%	100.00%	2.91	2.67	70.54%	0.00%	100.00%	2.20	2.67	60.04%	11.90%	1.16	0.04	208.21	9.04	31.13	0.83
NPHP4	1	9	59	59	59	2.20	1.00	19.19%	0.00%	0.00%	0.00	0.00			55.56%	0.04	0.44	64.42%	0.00%	100.00%	6.05	1.67	57.05%	60.07%	1.24	0.04	225.33	7.56	204.69	0.70
NR2E3	27	384	9	40	70	1.94	1.02	10.88%	7.39%	31.51%	1.42	0.76	10.23%	12.89%	21.35%	0.37	0.50	56.46%	17.86%	39.58%	1.72	1.40	52.18%	24.68%	1.32	0.08	202.83	5.85	77.48	0.72
NR2F1	1	5	22	29	32	1.87	1.00	11.70%	0.00%	0.00%	0.00	0.00			0.00%	0.00	0.00			0.00%	0.00	0.00			1.32	0.08	209.49	15.72	110.93	0.65
NRL	6	26	20	47	80	2.01	1.04	16.78%	8.27%	69.23%	8.00	2.19	13.37%	41.91%	26.92%	0.12	0.31	56.64%	88.18%	69.23%	2.80	1.85	48.05%	90.56%	1.16	0.03	201.74	8.27	54.69	0.68
NYX	7	46	5	15.5	46	2.39	1.00	13.23%	1.03%	4.35%	0.20	0.26	2.38%	57.84%	4.35%	0.00	0.04	65.70%	0.00%	13.04%	0.36	0.28	59.71%	40.38%	1.22	0.07	202.06	5.87	44.63	0.72
OAT	8	143	22	43.5	64	1.93	1.14	11.96%	1.96%	74.83%	47.65	2.17	9.98%	12.27%	61.54%	0.22	1.01	51.11%	20.33%	18.88%	0.12	0.31	35.56%	12.35%	1.13	0.03	191.12	7.10	85.81	0.67
OCA1	1	1	9	25.5	42	0.97	1.00	10.58%	0.00%	0.00%	0.00	0.00			0.00%	0.00	0.00			0.00%	0.00	0.00			0.93	0.02	189.77	2.07	30.82	0.52
OCA2	8	79	4	27	83	1.64	1.01	24.24%	1.33%	7.59%	0.99	0.16	27.76%	3.03%	3.80%	0.00	0.04	75.84%	0.00%	0.00%	0.00	0.00			1.05	0.02	202.58	6.34	42.42	0.65
OPA1	26	127	11	36	79	1.89	1.00	17.48%	5.24%	1.57%	0.01	0.02	12.34%	0.00%	16.54%	0.01	0.30	57.81%	8.61%	0.79%	0.00	0.01	68.94%	0.00%	1.40	0.10	210.36	5.59	71.17	0.73
OPN1LW	1	2	31	44	57	2.17	1.00	10.64%	0.00%	0.00%	0.00	0.00			50.00%	0.25	1.00	55.91%	0.00%	0.00%	0.00	0.00			1.46	0.12	181.40	3.60	39.21	0.74
PAX2	1	10	33	39	45	2.06	1.00	30.18%	0.00%	10.00%	1.97	0.10	24.27%	23.62%	10.00%	0.01	0.10	77.32%	0.00%	10.00%	0.00	0.10	74.14%	0.00%	1.12	0.03	207.97	4.36	28.24	0.53
PAX6	7	27	11	29	75	1.78	1.00	15.30%	0.00%	0.00%	0.00	0.00			7.41%	0.00	0.00	70.69%		0.00%	0.00	0.00			1.08	0.04	195.64	7.60	43.89	0.69
PCDH15	8	103	19	52	81	2.17	1.04	14.76%	0.00%	74.76%	9.68	2.16	10.91%	24.24%	33.01%	0.15	0.47	56.83%	62.04%	87.38%	4.50	3.33	51.23%	65.06%	1.12	0.02	162.39	6.92	98.70	0.67
PDE6A	13	108	22	36	85	2.61	1.02	16.48%	0.02%	52.78%	12.80	1.38	17.06%	23.69%	35.19%	0.19	0.51	49.45%	67.39%	65.74%	2.14	3.56	49.36%	95.54%	1.09	0.03	197.01	10.30	197.25	0.71
PDE6B	24	272	10	41.5	87	2.62	1.01	14.75%	3.35%	52.21%	4.48	0.93	17.05%	49.30%	21.32%	0.08	0.28	55.09%	56.89%	86.03%	3.37	4.06	47.35%	86.55%	1.12	0.03	181.70	7.22	113.36	0.71
PDE6C	7	66	9	43	58	2.17	1.00	14.32%	6.53%	40.91%	1.21	0.52	11.44%	79.35%	4.55%	0.00	0.03	47.63%	100.00%	45.45%	1.25	2.65	45.12%	68.89%	1.14	0.03	209.81	10.06	141.35	0.66
PDE6G	2	17	46	61.5	77	1.93	1.00	14.42%	0.00%	94.12%	24.60	2.06	8.28%	2.59%	5.88%	0.06	0.06	42.42%	100.00%	58.82%	0.89	0.88	52.89%	100.00%	0.93	0.01	146.18	8.57	97.44	0.63
PEX1	1	1	19	19	19	1.12	1.00	16.41%	0.00%	0.00%	0.00	0.00			0.00%	0.00	0.00			0.00%	0.00	0.00			0.96	0.01	169.76	5.39	20.10	0.79
PHYH	3	23	36	42	43	1.69	1.04	16.25%	7.65%	73.91%	15.09	2.00	16.61%	49.14%	17.39%	0.13	0.22	48.62%	70.92%	56.52%	2.32	1.57	52.79%	55.29%	1.02	0.02	211.91	19.76	587.68	0.70
PMM2	1	8	50	50	50	3.17	1.00	9.85%	0.00%	50.00%	0.51	0.50	16.16%	100.00%	0.00%	0.00	0.00			50.00%	1.49	1.50	43.02%	100.00%	1.32	0.07	189.42	3.90	34.53	0.72
PNPLA6	4	44	13	27	64	1.09	0.95	17.85%	37.45%	90.91%	76.83	2.05	16.65%	41.66%	11.36%	0.03	0.14	32.67%	60.43%	20.45%	1.52	3.11	45.99%	77.18%	1.11	0.03	212.07	12.77	169.49	0.70

gene	patient count	image count	Patient Ages			Disc				Hypo-AF					Hyper-AF					Ring					Vessels					
						area	num clusters	intensity	% <6mm	incidence	area	num clusters	intensity	% <6mm	incidence	area	num clusters	intensity	% <6mm	incidence	area	num clusters	intensity	% <6mm	Fractal Dimension	Density	Width	Distance tortuosity mean	Squared curvature tortuosity mean	Tortuosity density mean
**Total**	**3491**	**32964**	**min**	**median**	**max**	**2.09**	**1.02**	**13.51%**	**6.86%**	**61.15%**	**13.04**	**1.46**	**13.02%**	**48.17%**	**25.11%**	**0.15**	**0.43**	**57.14%**	**52.81%**	**43.71%**	**1.19**	**1.78**	**54.10%**	**77.92%**	**1.25**	**0.06**	**186.52**	**6.45**	**98.03**	**0.70**
POC1B	4	39	30	38	59	2.01	1.00	15.65%	6.44%	12.82%	0.80	0.13	24.00%	99.92%	2.56%	0.00	0.03	83.77%	0.00%	7.69%	0.17	0.41	58.99%	71.49%	1.33	0.08	213.24	8.42	149.54	0.73
PROM1	52	461	12	46	86	2.31	1.01	12.44%	6.86%	80.69%	12.22	1.73	12.48%	72.05%	20.61%	0.03	0.25	52.15%	72.15%	34.49%	0.91	2.02	53.90%	82.54%	1.26	0.06	180.39	6.32	113.25	0.72
PRPF3	6	68	19	42	87	2.19	0.99	19.71%	4.63%	73.53%	3.76	1.07	13.84%	67.07%	45.59%	0.61	0.50	56.20%	86.15%	76.47%	3.17	2.24	46.35%	72.56%	1.03	0.02	181.57	9.51	132.07	0.72
PRPF31	57	592	12	46	95	2.20	1.03	15.53%	1.24%	48.14%	6.78	1.56	11.97%	41.34%	18.75%	0.12	0.34	45.89%	68.33%	64.02%	2.17	2.85	52.53%	86.92%	1.21	0.04	178.26	7.11	146.19	0.70
PRPF6	4	13	45	58	59	2.41	1.00	21.36%	13.61%	76.92%	7.04	1.15	19.73%	16.43%	30.77%	0.11	0.69	46.10%	81.05%	53.85%	4.38	0.54	44.78%	40.87%	1.20	0.04	189.15	6.06	67.83	0.72
PRPF8	18	172	20	51	83	2.33	1.02	14.57%	2.46%	29.65%	1.73	0.65	10.35%	26.14%	13.95%	0.02	0.15	62.66%	44.31%	86.05%	2.78	2.72	52.30%	88.57%	1.12	0.03	167.83	10.67	145.07	0.69
PRPH2	148	1218	9	59	91	2.05	1.01	12.34%	4.47%	68.72%	10.28	1.89	10.10%	62.00%	36.37%	0.07	0.58	59.10%	77.08%	26.27%	0.69	0.85	55.77%	69.43%	1.36	0.08	184.62	4.76	52.09	0.71
PRSS56	1	2	30	30	30	1.98	1.00	22.54%	11.52%	0.00%	0.00	0.00			0.00%	0.00	0.00			0.00%	0.00	0.00			1.42	0.13	262.82	2.82	18.30	0.72
PYGM	3	14	50	73	80	2.21	1.00	17.53%	0.00%	21.43%	3.02	0.21	10.63%	82.58%	71.43%	0.09	1.14	62.44%	81.32%	0.00%	0.00	0.00			1.42	0.11	145.64	3.71	29.34	0.74
RAB28	1	32	12	12	12	2.63	1.00	11.14%	0.00%	0.00%	0.00	0.00			46.88%	0.13	0.47	72.50%	100.00%	100.00%	1.32	1.41	69.75%	100.00%	1.32	0.06	218.28	4.60	118.18	0.69
RAX2	3	40	45	49	70	2.58	1.00	20.76%	19.89%	90.00%	6.76	1.05	22.01%	52.94%	7.50%	0.02	0.10	62.30%	34.82%	32.50%	0.07	0.63	48.86%	77.67%	1.26	0.05	208.85	10.22	118.59	0.70
RBP3	2	17	25	28.5	32	2.06	1.00	15.14%	1.73%	70.59%	7.06	1.35	13.79%	34.18%	52.94%	0.67	1.53	45.94%	22.22%	11.76%	0.01	0.12	51.40%	50.00%	1.05	0.02	164.58	8.97	70.22	0.67
RDH12	29	279	9	36	67	1.92	1.01	18.02%	11.77%	58.06%	22.35	1.10	19.00%	37.32%	33.69%	0.37	0.52	49.05%	22.06%	34.05%	1.51	2.52	57.88%	84.76%	0.99	0.03	179.48	3.48	20.59	0.72
RDH5	9	76	13	50	84	1.21	1.04	27.31%	3.19E-05	22.37%	2.90	0.39	16.27%	17.58%	23.68%	0.14	0.38	44.14%	8.08%	19.74%	0.25	0.58	41.17%	40.00%	1.01	0.02	193.80	5.48	69.47	0.70
REEP6	3	43	39	44	62	2.45	1.02	18.13%	0.00%	44.19%	1.78	1.02	15.53%	15.83%	9.30%	0.01	0.12	61.28%	50.00%	90.70%	2.38	5.53	57.55%	97.92%	1.18	0.02	184.26	11.54	415.76	0.75
RGR	1	10	85	85	85	1.95	1.00	31.35%	0.00%	100.00%	111.10	3.50	33.96%	17.84%	0.00%	0.00	0.00			0.00%	0.00	0.00			1.14	0.02	218.95	6.17	66.37	0.73
RHO	107	968	9	50	95	2.17	1.03	15.53%	1.53%	61.88%	9.74	1.72	13.64%	34.76%	18.49%	0.11	0.24	52.95%	55.98%	70.56%	2.25	2.99	53.62%	82.80%	1.22	0.04	175.72	6.49	86.13	0.69
RLBP1	6	42	15	46.5	83	1.62	1.00	19.39%	0.30%	69.05%	7.51	1.90	16.27%	27.24%	33.33%	0.04	0.38	45.37%	63.77%	9.52%	0.04	0.12	40.72%	100.00%	1.11	0.03	209.37	19.39	327.98	0.69
RP1	115	951	18	59.5	93	2.19	1.01	13.69%	5.55%	62.99%	9.32	1.76	13.07%	40.06%	19.14%	0.18	0.27	55.90%	66.70%	64.98%	2.03	2.98	54.56%	74.81%	1.23	0.05	174.37	6.93	139.44	0.70
RP1L1	18	169	13	55	90	1.82	1.04	16.11%	7.92%	48.52%	3.52	1.76	4.43%	29.00%	8.28%	0.00	0.06	56.38%	44.95%	30.18%	0.79	1.15	54.11%	79.65%	1.30	0.08	200.64	4.74	46.77	0.66
RP2	28	332	11	31.5	81	2.48	1.05	9.87%	13.25%	47.59%	3.10	1.19	10.55%	48.28%	6.93%	0.02	0.08	54.74%	84.40%	12.95%	0.26	0.38	58.97%	67.79%	1.19	0.04	181.19	6.83	109.26	0.69
RP9	9	103	21	50	74	2.10	1.02	15.49%	1.94%	40.78%	3.50	1.51	11.09%	13.82%	7.77%	0.01	0.07	48.98%	50.03%	67.96%	3.34	1.70	54.39%	71.25%	1.24	0.05	171.05	6.24	121.52	0.69
RPE65	18	82	5	26	82	1.15	1.06	19.28%	7.57%	52.44%	40.13	2.65	15.03%	29.14%	37.80%	0.24	0.65	56.35%	61.75%	4.88%	0.19	0.05	43.10%	97.60%	0.97	0.02	164.35	6.64	110.77	0.65
RPGR	161	1429	7	44	87	2.31	1.01	13.72%	3.67%	54.23%	7.78	1.27	14.36%	36.54%	22.60%	0.13	0.31	54.42%	58.64%	61.30%	2.13	2.09	55.94%	81.99%	1.19	0.04	182.93	8.03	126.10	0.70
RPGRIP1	7	93	17	27.5	59	2.73	1.00	13.28%	0.00%	4.30%	0.00	0.04	7.57%	25.00%	15.05%	0.02	0.15	51.47%	0.00%	70.97%	1.08	3.42	43.87%	85.01%	1.16	0.03	206.41	9.28	284.07	0.71
RS1	100	1186	7	33	78	1.93	1.00	11.86%	3.50%	26.81%	2.41	0.58	10.68%	36.67%	24.79%	0.10	0.37	44.28%	73.17%	16.95%	0.35	0.59	53.00%	66.36%	1.34	0.09	207.50	5.45	62.27	0.72
SAG	4	47	15	47.5	58	2.99	1.04	13.28%	0.00%	31.91%	1.19	0.53	20.98%	63.99%	0.00%	0.00	0.00			14.89%	0.42	0.47	54.36%	89.84%	1.20	0.04	228.97	9.02	87.09	0.69
SDCCAG8	1	11	29	29	29	3.19	1.00	9.09%	0.00%	0.00%	0.00	0.00			0.00%	0.00	0.00			100.00%	1.43	3.91	51.65%	100.00%	1.40	0.08	182.97	7.54	83.15	0.76
SGSH	1	2	34	34	34	3.23	1.00	6.49%	0.00%	100.00%	2.46	2.50	6.39%	0.15%	0.00%	0.00	0.00			100.00%	1.38	3.00	40.38%	100.00%	1.32	0.05	188.58	6.61	32.76	0.73
SLC24A1	1	4	28	28	28	2.67	1.00	13.92%	0.00%	0.00%	0.00	0.00			75.00%	0.92	3.75	67.49%	0.00%	75.00%	1.77	1.75	50.43%	0.00%	1.46	0.11	179.34	4.31	51.46	0.73
SLC24A5	1	5	18	21	28	1.75	1.00	13.56%	0.00%	0.00%	0.00	0.00			0.00%	0.00	0.00			0.00%	0.00	0.00			1.36	0.10	242.22	10.66	162.99	0.76
SLC25A46	1	4	65	65	65	2.15	1.00	17.71%	0.00%	0.00%	0.00	0.00			0.00%	0.00	0.00			0.00%	0.00	0.00			1.44	0.11	195.15	6.97	144.31	0.71
SNRNP200	12	122	29	48	79	2.26	1.00	19.33%	0.20%	73.77%	18.05	2.05	15.78%	29.08%	30.33%	0.21	0.52	51.82%	69.42%	74.59%	2.13	3.57	49.38%	96.55%	1.13	0.03	166.44	6.64	88.10	0.69
SPATA7	2	20	21	35	57	2.54	1.15	13.78%	4.94%	5.00%	0.13	0.10	28.36%	0.00%	35.00%	0.05	0.35	62.23%	13.51%	90.00%	1.83	3.60	40.23%	90.97%	1.14	0.03	197.80	8.37	98.86	0.70
SSBP1	3	35	29	62	63	1.38	1.00	16.74%	18.76%	77.14%	0.62	2.49	8.56%	49.16%	20.00%	0.01	0.26	76.85%	0.00%	40.00%	1.41	1.86	54.33%	69.36%	1.20	0.04	165.92	5.26	92.93	0.73
TIMP3	36	322	26	57	78	1.71	1.01	17.51%	9.49%	62.11%	16.35	1.84	15.26%	46.54%	30.75%	0.14	0.51	58.46%	32.56%	27.02%	0.47	1.00	55.24%	35.32%	1.39	0.09	198.27	4.99	71.86	0.71
TOPORS	6	83	20	52.5	72	2.16	0.99	13.72%	0.00%	75.90%	2.58	1.88	17.39%	80.33%	4.82%	0.01	0.05	70.07%	25.00%	93.98%	3.05	4.57	56.99%	81.22%	1.29	0.06	187.25	6.93	80.81	0.71
TRNT1	1	10	41	41	41	2.62	1.00	15.62%	0.00%	10.00%	0.00	0.00	13.96%	0.00%	0.00%	0.00	0.00			100.00%	5.69	3.60	70.63%	94.80%	1.15	0.02	184.94	10.53	269.64	0.68
TRPM1	6	27	8	21	57	2.65	1.00	14.22%	0.67%	0.00%	0.00	0.00			0.00%	0.00	0.00			0.00%	0.00	0.00			1.24	0.06	207.28	7.28	63.60	0.66
TSPAN12	1	1	23	30.5	38	2.16	1.00	9.87%	0.00%	0.00%	0.00	0.00			0.00%	0.00	0.00			0.00%	0.00	0.00			1.47	0.13	205.51	4.79	64.06	0.71
TTLL5	8	56	37	47	70	2.34	1.00	14.86%	1.59%	85.71%	3.36	1.68	17.98%	95.01%	30.36%	0.03	0.27	37.89%	100.00%	100.00%	3.07	5.32	50.65%	88.34%	1.32	0.06	213.57	8.00	121.73	0.76
TULP1	16	165	7	33	69	2.57	1.05	13.77%	2.78%	26.06%	0.53	0.41	15.25%	63.18%	21.82%	0.07	0.40	52.38%	27.71%	78.18%	2.56	2.96	47.46%	75.10%	1.15	0.04	199.61	12.24	251.64	0.70
TYR	12	91	5	34	76	1.63	1.00	12.26%	12.36%	3.30%	0.01	0.14	17.37%	16.77%	9.89%	0.01	0.20	63.87%	26.92%	0.00%	0.00	0.00			1.37	0.09	217.99	5.12	69.35	0.72
TYRP1	1	4	13	19.5	26	1.87	1.00	12.90%	0.00%	0.00%	0.00	0.00			0.00%	0.00	0.00			0.00%	0.00	0.00			1.46	0.13	208.11	3.89	32.71	0.73
USH1C	14	99	22	41	81	2.05	1.01	19.20%	2.54%	66.67%	7.01	1.63	11.29%	34.47%	29.29%	0.60	0.56	52.39%	41.64%	46.46%	1.83	1.59	46.45%	62.73%	1.01	0.02	167.35	14.95	516.55	0.67
USH1G	2	12	32	38.5	45	1.89	1.00	13.40%	11.93%	33.33%	0.20	0.42	9.90%	0.00%	50.00%	0.33	1.08	56.63%	0.00%	58.33%	2.89	5.00	58.58%	1.79%	1.23	0.04	234.70	8.28	94.58	0.73
USH2A	306	2858	0	52	91	2.17	1.02	15.63%	2.32%	61.30%	8.47	1.76	12.99%	36.45%	19.84%	0.15	0.31	53.46%	66.47%	72.08%	1.98	3.20	53.47%	87.51%	1.17	0.03	170.22	7.26	101.60	0.69
USH2C	5	59	47	56	61	1.69	0.98	16.12%	3.42%	88.14%	10.99	2.12	11.67%	20.83%	32.20%	0.24	0.47	58.05%	90.79%	79.66%	2.09	2.98	52.11%	88.24%	1.07	0.02	156.45	6.12	82.23	0.70
VHL	1	2	27	27	27	2.78	1.00	20.14%	0.00%	0.00%	0.00	0.00			0.00%	0.00	0.00			0.00%	0.00	0.00			1.40	0.10	223.00	6.09	43.62	0.71
VPS13B	3	37	34	35	65	2.10	1.00	25.51%	0.00%	48.65%	1.62	0.57	19.57%	85.24%	18.92%	0.04	0.22	43.57%	4.28%	70.27%	1.74	1.30	49.23%	94.07%	0.85	0.01	172.54	9.77	192.76	0.76
WDR19	2	27	18	31	44	2.57	1.00	12.46%	0.00%	22.22%	2.93	0.41	14.56%	92.55%	11.11%	0.00	0.11	30.38%	92.59%	96.30%	1.55	2.37	57.59%	20.26%	1.37	0.07	162.48	3.79	22.94	0.75
WFS1	4	21	18	32.5	79	1.87	1.00	24.03%	1.16%	0.00%	0.00	0.00			19.05%	0.01	1.10	62.50%	21.98%	0.00%	0.00	0.00			1.40	0.10	208.53	4.40	52.44	0.74
