# Supplementary material for: Quantification of Fundus Autofluorescence Features in a Molecularly Characterized Cohort of >3500 Patients with Inherited Retinal Disease from the United Kingdom
Source: Ophthalmol Sci. 2024 Nov 12;5(2):100652. doi: 10.1016/j.xops.2024.100652 (PMC11782848; doi:10.1016/j.xops.2024.100652)
Supplement: Table S5 [file mmc14.pdf]

Table S5: Feature statistics by gene for selected 30 genes. Results are mean across all images. %=Incidence, A=Average Area (mm^2), C=Num Components, I = Intensity (pixel brightness), <3mm=Proportion of feature area within 3mm of the fovea (corresponding to outer 6mm ETRDS ring), D=vessel density, F=fractal dimension. The table cells have been shaded with lower values in red, intermediate values in white and larger values in green.

	disc				hypo-AF					hyper-AF					ring					vessels	
**Gene**	**A**	**C**	**I**	**<3mm**	**%**	**A**	**C**	**I**	**<3mm**	**%**	**A**	**C**	**I**	**<3mm**	**%**	**A**	**C**	**I**	**<3mm**	**D**	**F**
ABCA4	2.19	1.01	9.20%	13.70%	80.30%	19.81	1.45	11.10%	61.30%	18.00%	0.05	0.33	66.40%	48.30%	33.50%	0.57	1.58	59.60%	81.80%	7.65%	1.33
ABCC6	1.53	1.03	16.90%	17.60%	69.00%	21.44	2.08	14.30%	32.60%	54.90%	0.23	1.2	62.50%	23.40%	8.20%	0.03	0.15	43.30%	38.00%	8.07%	1.37
BBS1	2.59	1.02	10.40%	7.50%	67.50%	8.35	1.29	11.90%	56.90%	25.90%	0.15	0.38	47.30%	86.90%	32.50%	0.84	1.03	56.50%	72.70%	4.56%	1.18
BEST1	1.84	1.01	11.60%	4.10%	40.50%	2.58	0.9	11.50%	59.70%	57.50%	0.73	1.09	61.20%	59.80%	57.50%	1.28	1.83	56.30%	77.20%	9.62%	1.38
CACNA1F	2.04	1.01	14.80%	0.00%	5.70%	0.91	0.2	7.10%	42.40%	12.80%	0.01	0.15	59.80%	2.50%	3.80%	0.01	0.06	53.60%	79.80%	5.35%	1.21
CDH23	2.05	1.01	14.30%	6.40%	16.20%	0.71	0.47	10.40%	42.00%	19.60%	0.23	0.5	57.60%	34.20%	77.30%	2.46	2.73	47.30%	67.30%	2.46%	1.05
CERKL	2.63	1	12.30%	9.10%	82.30%	10.33	1.43	12.10%	51.70%	17.70%	0.02	0.21	46.00%	88.60%	29.70%	1.04	0.95	58.10%	65.30%	3.51%	1.16
CHM	1.45	1.06	20.60%	2.80%	82.60%	51.77	3.28	19.90%	21.00%	48.80%	0.26	0.83	54.50%	37.90%	5.50%	0.03	0.08	43.20%	32.20%	6.20%	1.3
CNGA3	1.74	1.01	13.80%	14.50%	31.10%	1.48	0.96	7.10%	56.30%	8.00%	0.05	0.17	54.00%	31.80%	35.60%	0.32	0.75	50.00%	74.90%	5.36%	1.24
CNGB3	1.91	0.99	14.90%	12.70%	12.50%	0.16	0.38	6.70%	73.60%	10.40%	0.01	0.12	52.70%	63.90%	32.60%	0.2	0.63	54.60%	92.20%	5.92%	1.24
CRB1	1.97	1.02	14.70%	3.60%	55.20%	8.3	1.22	15.60%	40.40%	44.00%	0.41	0.89	46.40%	54.00%	43.70%	1.73	1.35	48.90%	59.20%	4.27%	1.11
CRX	2.11	1	15.60%	5.70%	70.30%	5.51	1.39	15.60%	62.70%	16.90%	0.05	0.26	50.70%	29.90%	59.30%	2.23	4.22	52.90%	65.90%	7.30%	1.33
EFEMP1	1.77	1	15.30%	6.00%	78.70%	5.15	1.84	14.20%	82.40%	77.90%	0.24	1.55	61.80%	79.40%	24.10%	0.18	0.66	60.00%	82.50%	10.10%	1.43
EYS	2.36	1.02	15.20%	2.90%	65.90%	9.55	1.64	13.30%	27.30%	23.40%	0.23	0.4	57.60%	42.90%	72.50%	2.08	2.76	52.60%	76.80%	3.07%	1.16
GUCY2D	2	1	14.70%	6.50%	48.00%	5.25	0.86	17.50%	71.20%	17.60%	0.22	0.3	61.20%	36.00%	60.90%	1.51	2.94	53.10%	75.00%	6.91%	1.29
MYO7A	2.07	0.99	15.80%	2.30%	42.30%	2.37	0.95	18.00%	32.90%	28.00%	0.41	0.97	53.70%	21.80%	73.60%	3.24	3.26	47.00%	57.80%	2.70%	1.09
NR2E3	1.94	1.02	10.90%	7.40%	31.50%	1.42	0.76	10.20%	12.90%	21.40%	0.37	0.5	56.50%	17.90%	39.60%	1.72	1.4	52.20%	24.70%	8.33%	1.32
PDE6B	2.62	1.01	14.80%	3.40%	52.20%	4.48	0.93	17.00%	49.30%	21.30%	0.08	0.28	55.10%	56.90%	86.00%	3.37	4.06	47.30%	86.60%	3.00%	1.12
PROM1	2.3	1.01	12.60%	7.00%	80.00%	12.15	1.72	12.60%	72.00%	21.10%	0.04	0.25	52.30%	72.90%	34.80%	0.85	2.06	54.00%	82.50%	6.10%	1.26
PRPF31	2.2	1.03	15.50%	1.20%	48.10%	6.78	1.56	12.00%	41.30%	18.80%	0.12	0.34	45.90%	68.30%	64.00%	2.17	2.85	52.50%	86.90%	4.10%	1.21
PRPH2	2.05	1.01	12.30%	4.50%	68.70%	10.28	1.89	10.10%	62.00%	36.40%	0.07	0.58	59.10%	77.10%	26.30%	0.69	0.85	55.80%	69.40%	8.00%	1.36
RDH12	1.92	1.01	18.00%	11.80%	58.10%	22.35	1.1	19.00%	37.30%	33.70%	0.37	0.52	49.00%	22.10%	34.10%	1.51	2.52	57.90%	84.80%	2.93%	0.99
RHO	2.17	1.03	15.50%	1.50%	61.90%	9.74	1.72	13.60%	34.80%	18.50%	0.11	0.24	53.00%	56.00%	70.60%	2.25	2.99	53.60%	82.80%	4.47%	1.22
RP1	2.19	1.01	13.70%	5.50%	63.00%	9.32	1.76	13.10%	40.10%	19.10%	0.18	0.27	55.90%	66.70%	65.00%	2.03	2.98	54.60%	74.80%	4.59%	1.23
RP2	2.48	1.05	9.90%	13.30%	47.60%	3.1	1.19	10.50%	48.30%	6.90%	0.02	0.08	54.70%	84.40%	13.00%	0.26	0.38	59.00%	67.80%	3.65%	1.19
RPE65	1.15	1.06	19.30%	7.60%	52.40%	40.13	2.65	15.00%	29.10%	37.80%	0.24	0.65	56.40%	61.80%	4.90%	0.19	0.05	43.10%	97.60%	2.04%	0.97
RPGR	2.31	1.01	13.70%	3.70%	54.20%	7.78	1.27	14.40%	36.50%	22.60%	0.13	0.31	54.40%	58.60%	61.30%	2.13	2.09	55.90%	82.00%	4.06%	1.19
RS1	1.93	1	11.90%	3.50%	26.80%	2.41	0.58	10.70%	36.70%	24.80%	0.1	0.37	44.30%	73.20%	16.90%	0.35	0.59	53.00%	66.40%	8.69%	1.34
TIMP3	1.71	1.01	17.50%	9.50%	62.10%	16.35	1.84	15.30%	46.50%	30.70%	0.14	0.51	58.50%	32.60%	27.00%	0.47	1	55.20%	35.30%	9.15%	1.39
USH2A	2.17	1.02	15.60%	2.30%	61.30%	8.47	1.76	13.00%	36.50%	19.80%	0.15	0.31	53.50%	66.50%	72.10%	1.98	3.2	53.50%	87.50%	3.42%	1.17
**All**	**2.09**	**1.02**	**13.50%**	**6.90%**	**61.20%**	**13.04**	**1.46**	**13.00%**	**48.20%**	**25.10%**	**0.15**	**0.43**	**57.10%**	**52.80%**	**43.70%**	**1.19**	**1.78**	**54.10%**	**77.90%**	**5.75%**	**1.25**
