# Supplementary material for: Quantification of Fundus Autofluorescence Features in a Molecularly Characterized Cohort of >3500 Patients with Inherited Retinal Disease from the United Kingdom
Source: Ophthalmol Sci. 2024 Nov 12;5(2):100652. doi: 10.1016/j.xops.2024.100652 (PMC11782848; doi:10.1016/j.xops.2024.100652)
Supplement: Table S7 [file mmc16.pdf]

Table S7: All vessel metrics across selected genes. Definitions of metrics are given in **Table S3** . The table cells have been shaded with lower values in red, intermediate values in white and larger values in green.

gene	Fractal Dimension	Vessel Density	Average Width	Distance Tortuosity Mean	Squared Curvature Tortuosity Mean	Tortuosity Density Mean
ABCA4	1.33	7.65%	184.81	4.81	61.6	0.71
ABCC6	1.37	8.07%	196.68	6.17	80.7	0.73
BBS1	1.18	4.56%	186.86	9.66	214.9	0.7
BEST1	1.38	9.62%	203.18	4.46	49.4	0.71
CACNA1F	1.21	5.35%	218.03	9.24	83.8	0.73
CDH23	1.05	2.46%	198.91	7.97	177.8	0.68
CERKL	1.16	3.51%	179.55	7.2	95.8	0.67
CHM	1.3	6.20%	190.34	6.14	104.4	0.69
CNGA3	1.24	5.36%	209.19	5.17	70.9	0.7
CNGB3	1.24	5.92%	205.53	6.42	67.1	0.7
CRB1	1.11	4.27%	178.82	7.2	83.2	0.69
CRX	1.33	7.30%	192.4	9.58	119.5	0.72
EFEMP1	1.43	10.06%	208.55	4.31	39.4	0.72
EYS	1.16	3.07%	172.31	7.39	108.3	0.69
GUCY2D	1.29	6.91%	197.87	5.41	64.4	0.72
MYO7A	1.09	2.70%	182.09	11	296.8	0.7
NR2E3	1.32	8.33%	202.83	5.85	77.5	0.72
PDE6B	1.12	3.00%	181.7	7.22	113.4	0.71
PROM1	1.26	6.10%	179.6	6.5	116.9	0.72
PRPF31	1.21	4.10%	178.26	7.11	146.2	0.7
PRPH2	1.36	8.00%	184.62	4.76	52.1	0.71
RDH12	0.99	2.93%	179.48	3.48	20.6	0.72
RHO	1.22	4.47%	175.72	6.49	86.1	0.69
RP1	1.23	4.59%	174.37	6.93	139.4	0.7
RP2	1.19	3.65%	181.19	6.83	109.3	0.69
RPE65	0.97	2.04%	164.35	6.64	110.8	0.65
RPGR	1.19	4.06%	182.93	8.03	126.1	0.7
RS1	1.34	8.69%	207.5	5.45	62.3	0.72
TIMP3	1.39	9.15%	198.27	4.99	71.9	0.71
USH2A	1.17	3.42%	170.22	7.26	101.6	0.69
**All**	**1.25**	**5.75%**	**186.52**	**6.45**	**98**	**0.7**
