# Supplementary material for: Quantification of Fundus Autofluorescence Features in a Molecularly Characterized Cohort of >3500 Patients with Inherited Retinal Disease from the United Kingdom
Source: Ophthalmol Sci. 2024 Nov 12;5(2):100652. doi: 10.1016/j.xops.2024.100652 (PMC11782848; doi:10.1016/j.xops.2024.100652)
Supplement: Table S8 [file mmc17.pdf]

Table S8: Average increase in hypo-AF area stratified by ABCA4 variant severity. ABCA4 patients are grouped based on the severity of their genetic variants as proposed by Cornelis et al. 2022 into groups A, B and C [27].

ABCA4 severity classification	Number of Patients	Variant combination	Average increase in hypo-AF area per year (mm^2)	Average increase in sqrt hypo-AF area per year (mm)
A	69	Severe/Severe	3.11	0.29
B	75	Intermediate/Intermediate or Severe/Intermediate	1.59	0.17
C	184	Mild/*	0.87	0.11
